# Impact of Internet-Based Interventions on Caregiver Mental Health: Systematic Review and Meta-Analysis

**DOI:** 10.2196/10668

**Published:** 2018-07-03

**Authors:** Diana Sherifali, Muhammad Usman Ali, Jenny Ploeg, Maureen Markle-Reid, Ruta Valaitis, Amy Bartholomew, Donna Fitzpatrick-Lewis, Carrie McAiney

**Affiliations:** ^1^ School of Nursing Faculty of Health Sciences McMaster University Hamilton, ON Canada; ^2^ McMaster Evidence Review and Synthesis Team School of Nursing, Faculty of Health Sciences McMaster University Hamilton, ON Canada; ^3^ Diabetes Care and Research Program Hamilton Health Sciences Hamilton, ON Canada; ^4^ Department of Health Research Methods, Evidence and Impact Faculty of Health Sciences McMaster University Hamilton, ON Canada; ^5^ Aging, Community and Health Research Unit School of Nursing, Faculty of Health Sciences McMaster University Hamilton, ON Canada; ^6^ Department of Health, Aging and Society Faculty of Social Sciences McMaster University Hamilton, ON Canada; ^7^ McMaster Institute for Research on Aging McMaster University Hamilton, ON Canada; ^8^ Person-Centred Interventions for Older Adults with Multimorbidity and their Caregivers Hamilton, ON Canada; ^9^ World Health Organization Collaborating Centre for Primary Care and Health Human Resources School of Nursing, Faculty of Health Sciences McMaster University Hamilton, ON Canada; ^10^ Department of Family Medicine McMaster University Hamilton, ON Canada; ^11^ Program for Interprofessional Practice, Education and Research Department of Psychiatry and Behavioural Neurosciences, Faculty of Health Sciences McMaster University Hamilton, ON Canada

**Keywords:** internet, support, education, mental health, caregivers, chronic conditions

## Abstract

**Background:**

The health of informal caregivers of adults with chronic conditions is increasingly vital since caregivers comprise a large proportion of supportive care to family members living in the community. Due to efficiency and reach, internet-based interventions for informal caregivers have the potential to mitigate the negative mental health outcomes associated with caregiving.

**Objective:**

The objective of this systematic review and meta-analysis was to examine the impact of internet-based interventions on caregiver mental health outcomes and the impact of different types of internet-based intervention programs.

**Methods:**

MEDLINE, EMBASE, CINAHL, PsycINFO, Cochrane, and AgeLine databases were searched for randomized controlled trials or controlled clinical trials published from January 1995 to April 2017 that compared internet-based intervention programs with no or minimal internet-based interventions for caregivers of adults with at least 1 chronic condition. The inclusion criteria were studies that included (1) adult informal caregivers (aged 18 years or older) of adults living in the community with a chronic condition; (2) an internet-based intervention program to deliver education, support, or monitoring to informal caregivers; and (3) outcomes of mental health. Title and abstract and full-text screening were completed in duplicate. Data were extracted by a single reviewer and verified by a second reviewer, and risk of bias assessments were completed accordingly. Where possible, data for mental health outcomes were meta-analyzed.

**Results:**

The search yielded 7923 unique citations of which 290 studies were screened at full-text. Of those, 13 studies met the inclusion criteria; 11 were randomized controlled trials, 1 study was a controlled clinical trial, and 1 study comprised both study designs. Beneficial effects of any internet-based intervention program resulted in a mean decrease of 0.48 points (95% CI –0.75 to –0.22) for stress and distress and a mean decrease of 0.40 points (95% CI –0.58 to –0.22) for anxiety among caregivers. For studies that examined internet-based information and education plus professional psychosocial support, the meta-analysis results showed small to medium beneficial effect sizes of the intervention for the mental health outcomes of depression (–0.34; 95% CI –0.63 to –0.05) and anxiety (–0.36; 95% CI –0.66 to –0.07). Some suggestion of a beneficial effect on overall health for the use of information and education plus combined peer and professional support was also shown (1.25; 95% CI 0.24 to 2.25). Overall, many studies were of poor quality and were rated at high risk of bias.

**Conclusions:**

The review found evidence for the benefit of internet-based intervention programs on mental health for caregivers of adults living with a chronic condition, particularly for the outcomes of caregiver depression, stress and distress, and anxiety. The types of interventions that predominated as efficacious included information and education with or without professional psychological support, and, to a lesser extent, with combined peer and psychological support. Further high-quality research is needed to inform the effectiveness of interactive, dynamic, and multicomponent internet-based interventions.

**Trial Registration:**

PROSPERO CRD42017075436; https://www.crd.york.ac.uk/prospero/display_record.php?RecordID=75436 (Archived by WebCite at http://www.webcitation.org/709M3tDvn)

## Introduction

The number of adults living with chronic conditions is increasing globally [1]. Many adults with chronic conditions rely on family or friend caregivers for support [2]. In Canada, it is reported that more than one-quarter of individuals provided support and care for a friend or family member with a long-term health condition, disability, or age-associated issue in a given year [3]. Caregivers supporting family members living with chronic conditions who need assistance with day-to-day functioning play an essential role for families but also for the health care system, as they provide up to 90% of the medical and supportive care needs for their care recipients [4,5]. While there are many benefits to caregiving for a family member, there are also detrimental emotional and mental health impacts of caregiving that are increasingly being identified and for which practical solutions are urgently needed [3,6,7].

Recognizing the negative health impacts of caregiving has led to studies to examine effective interventions to support these individuals. While a variety of interventions have been evaluated for their impact on improving the health of caregivers, with beneficial effects [8], there is great interest in the use of technology as a means of achieving positive outcomes. Eysenbach [9] suggests that efficiency of health care delivery through internet interventions may lead to a reduction in health care costs. Further, internet and eHealth may be more accessible to caregivers, especially those in remote and rural areas, resulting in increased equity to access health care [9].

There have been 15 recent systematic or other reviews of technology interventions (eg, internet, telephone) to support informal caregivers of adults with chronic conditions in the community [10-24]. Eight reviews focused on internet-based interventions designed specifically for caregivers [12,17,19-24]. All of these reviews provided evidence of improvements in caregivers’ health as a result of internet-based programs. A recent rapid evidence review evaluated the impact of internet-based interventions on outcomes for caregivers of persons with chronic conditions living in the community [24]. Internet-based interventions resulted in positive effects on mental health outcomes including decreasing depressive symptoms, stress or distress, and anxiety [24]. Limitations of these studies were that a meta-analysis was not performed to quantify the magnitude of effect across studies and determine clinical relevance; therefore, the impact of internet-based interventions on mental health outcomes of caregivers is still not clear.

The primary objective of this study was to conduct a systematic review and meta-analysis to assess the impact of internet-based interventions of any type compared to no or minimal internet-based interventions on the mental health of informal caregivers of adults with at least 1 chronic condition living in the community. The secondary objective was to examine whether specific types of internet-based interventions had a beneficial effect on caregiver mental health.

## Methods

This systematic review and meta-analysis was conducted following the Preferred Reporting Items for Systematic Reviews and Meta-analyses (PRISMA) guidelines [25].

### Population

The population of interest included informal caregivers aged 18 years and older who were currently providing caregiving support to adults aged 18 years and older (ie, care recipients) living in the community with at least 1 chronic condition.

### Interventions

Studies selected for this systematic review included those that examined any internet-based modality to deliver an intervention, which could include either a single component program or multimodal program to informal caregivers. An internet-based program was defined as any Web-based series of instructions, options, plans, lessons, modules, or curricula.

### Outcomes

The primary outcome of interest for this systematic review was mental health, specifically including depressive symptoms, stress/distress, anxiety, coping, overall mental health, quality of life, and overall health. A second paper on other caregiver outcomes reported in these studies (eg, self-efficacy, self-esteem, burden) is in progress.

### Study Design

#### Selection Criteria

Studies were included according to the following inclusion criteria: (1) study designs were a randomized controlled trial (RCT) or a controlled clinical trial (CCT), (2) studies examined any internet-based intervention program for informal caregivers of older adults having at least 1 chronic condition living in the community, (3) studies were published between January 1, 1995, and April 19, 2017, (4) studies were published in English, (5) studies reported on at least 1 mental health outcome of interest, (6) studies used any measurement tool to examine the mental health outcomes of interest, and (7) studies in which the comparator or control group received none or minimal internet-based intervention (eg, links to a website for information). There were no restrictions on the nature of chronic conditions of care recipients. Exclusion criteria included all other types of study designs (ie, observational studies, case reports), studies that compared different types of program- or module-specific internet-based interventions, grey or unpublished literature, conference abstracts, and letters or editorials. All study protocols without preliminary results for data extraction were also excluded.

#### Search Strategy

A peer-reviewed search strategy was developed by 2 research librarians at McMaster University. EMBASE, MEDLINE, PsycINFO, CINAHL, Cochrane, and AgeLine databases were searched for studies published between January 1, 1995 and April 19, 2017. Reference lists of systematic reviews were searched for relevant studies not captured by the initial search. Results were deduplicated, and the citations were uploaded to a secure internet-based platform. More detailed information about the search terms is available in Multimedia Appendix 1.

#### Selection of Studies

Two reviewers independently selected studies for possible inclusion based on title and abstract review. Studies deemed to have met inclusion criteria by either reviewer then underwent full-text review. Any disagreements were discussed between reviewers, and a third party was involved to help reach consensus as necessary.

### Data Extraction and Quality Assessment

Full data extraction, including characteristics of included studies, was completed by 1 reviewer and verified by a second reviewer. Risk of bias found in individual studies was assessed by 1 reviewer and verified by a second reviewer. Risk of bias was assessed using the Cochrane risk of bias framework [26], which evaluates the level of bias for sequence generation, allocation concealment, blinding, completeness of outcome assessment, selective reporting, and other biases. The quality of the clinical evidence was critically appraised by 1 reviewer and verified by a second reviewer using the Grading of Recommendations Assessment, Development, and Evaluation system (GRADE), which evaluates the risk for bias, inconsistency, indirectness, and imprecision for each outcome [27]. Disagreements were resolved through consensus between the 2 reviewers.

### Data Analysis

A meta-analysis was used to combine the results across studies by outcome using the published data from included studies. To perform the meta-analysis, we used immediate posttreatment data (mean, SD) for continuous outcomes such as depression, stress or distress, anxiety, coping, overall mental health, quality of life, and overall health. We used intention-to-treat outcome data where possible; however, if no intention-to-treat data were reported, we used study completer’s outcome data.

The DerSimonian and Laird random effects models with inverse variance method were used to generate the summary measures of effect in the form of standardized mean difference (SMD) [28]. The SMD accounts for similar outcomes measured using different assessment tools (eg, depressive symptoms were assessed using different outcome measures such as Center for Epidemiologic Studies Depression Scale and Beck Depression Inventory) [29]. In this situation, it was necessary to standardize the results of the studies to a uniform scale before they could be combined in a quantitative synthesis. SMDs were calculated using change from baseline data for intervention and control groups for each study with relevant outcome data. For each outcome, data from the corresponding study were used to calculate the mean difference between pretreatment (baseline) and posttreatment (final or end point) values along with its SD for both intervention and control groups. In studies where the SD was not reported, we calculated the SD from the reported standard error (SE) of the mean, 95% confidence intervals (CIs) and *P* values or *z* scores using equations provided in Chapter 7 and Chapter 9 of the Cochrane Handbook for Systematic Reviews of Interventions [30,31]. The SMD is interpreted based on its magnitude according to Cohen *d* recommended thresholds (~0.2=small effect, ~0.5=medium effect, ~0.8=large effect) [32].

The primary meta-analysis was to examine any type of internet-based intervention program by mental health outcome. The secondary meta-analysis was to examine the effects of specific types of internet-based intervention programs on mental health outcomes. Based on our previous work [24], intervention types were categorized accordingly: (1) internet-based information or education only, (2) internet-based information or education plus peer psychosocial support (PPS), (3) internet-based information or education plus professional psychosocial support (PFPS), (4) internet-based information or education plus combined peer and professional psychosocial support, and (5) internet-based intervention with telephone monitoring along with combined peer and professional psychosocial support.

Statistical heterogeneity of combined studies was examined using standard methods. The I^2^ statistic was used to quantify the magnitude of statistical heterogeneity between studies, where I^2^ of 30% to 60% represents moderate and I^2^ of >60% represents substantial heterogeneity [33]. A *P* value of <.10 was used as a guide to indicate where statistically significant heterogeneity may exist, upon which a closer examination of study differences was performed. All analyses were performed using the software packages Review Manager (RevMan version 5.3; The Cochrane Collaboration), STATA version 14 (StataCorp LLC), and GRADEpro Guideline Development Tool.

## Results

### Study Selection

**Figure 1:**
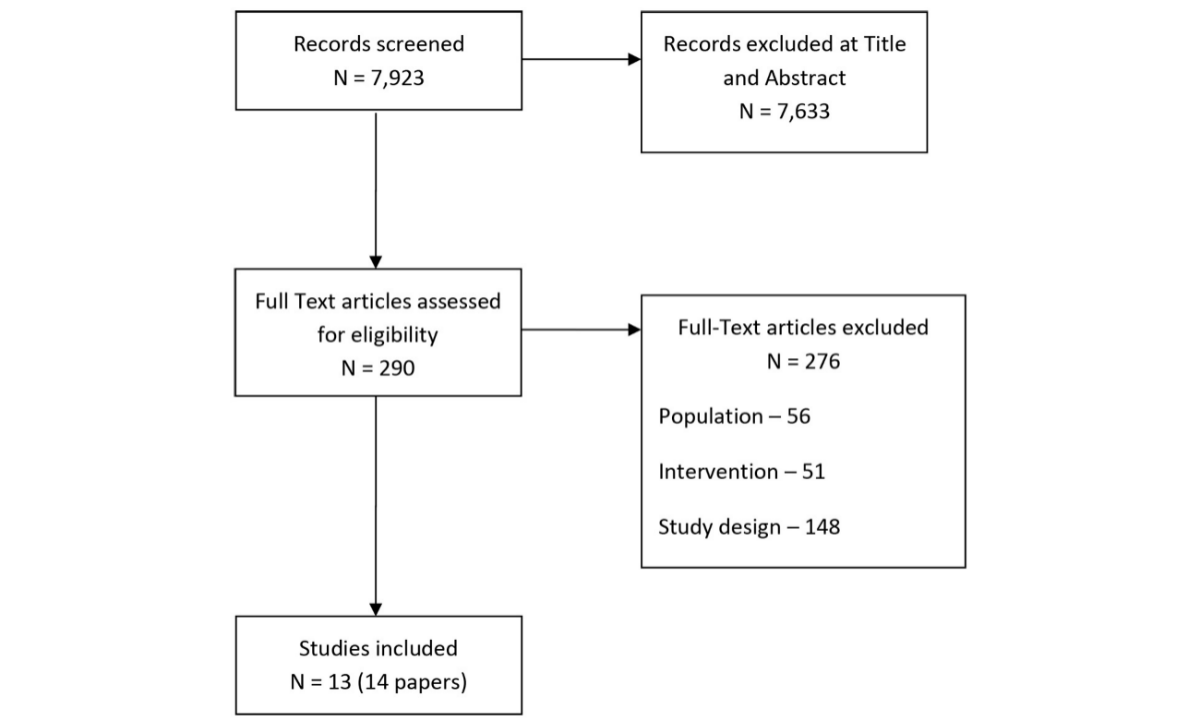
Study flowchart.

The search resulted in 7923 unique citations that were screened independently by 2 project staff. At title and abstract screening, we excluded 7633 studies, leaving 290 studies to be screened at full-text. Of those 290 studies, we identified 13 studies (14 papers) that met the inclusion criteria for this systematic review. References lists of the on-topic systematic reviews and included studies were searched but no additional studies were added (Figure 1).

### Description of Studies

The purpose, methods, participants, intervention, and risk of bias details of the included studies are shown in Multimedia Appendix 2. From among the 13 included studies, there were 11 studies that were RCTs [34-45], 1 study that was a CCT [46], and 1 study that combined both RCT and CCT designs [47]. Five of the included RCTs were conducted in Europe [34-38], and 5 RCTs were conducted in the United States [41-43,45], of which 1 RCT reported relevant outcomes across 2 papers [39,40]. There was 1 RCT conducted in Canada [44]. The CCT was conducted across the United States, Puerto Rico, and Mexico [46], and the combined CCT and RCT was conducted across 3 European countries [47].

In regard to the type of chronic conditions among care recipients, 9 studies included patients with some form of dementia [34-38,41,45-47]. Cardiovascular health disorders were represented in 3 studies, of which 2 studies included only stroke survivors [42,43] and the other study included a mixed stroke population of stroke-related dementia combined with patients having Alzheimer disease and Parkinson disease [44]. One study was based on non–small cell lung cancer care recipients [39,40]. All included studies were considered small in sample size (≤150 subjects per arm) and had a short length of study follow-up (<6 months). One study included a slightly longer study follow-up time period of 1-year [43]. A majority of studies included informal caregivers aged older than 50 years (range 53.8 to 67.8 years) [35-44,47], except in 1 study that included family caregivers who were also partially working and therefore reported a slightly lower age [45]. Two studies did not provide information on the average age of caregivers [34,46]. More than half of the caregivers were female in all of the included studies (range 56.3% to 100%).

From among the 13 included studies, there were 2 studies (15%) that were categorized as having used an internet-based information or education only intervention [41,45], 3 studies (23%) having used an internet-based information or education plus PPS intervention [34,36,37], 1 study (8%) having used an internet-based information or education plus PFPS intervention [35], 6 studies (46%) having used an internet-based information or education plus combined peer and professional psychosocial support intervention [38-40,42-44,46], and 1 study (8%) having used an internet-based intervention with telephone monitoring along with combined peer and professional psychosocial support [47].

Studies had a comparison group defined as receiving no internet-based intervention that could have included minimal guidance on information resources or website use [34,37,38,41,42,44], usual care with or without additional information [36,39,40,43,45,47], printed information [46], or electronic communications (eg, e-bulletins) [35].

Outcome assessment tools used for relevant mental health outcomes varied across studies and are summarized in Multimedia Appendix 3. Among the 13 included studies, outcomes examined included depression (8/13), stress or distress (6/13), anxiety (2/13), coping (2/13), overall mental health (1/13), quality of life (4/13), and overall health (2/13).

### Risk of Bias

**Figure 2:**
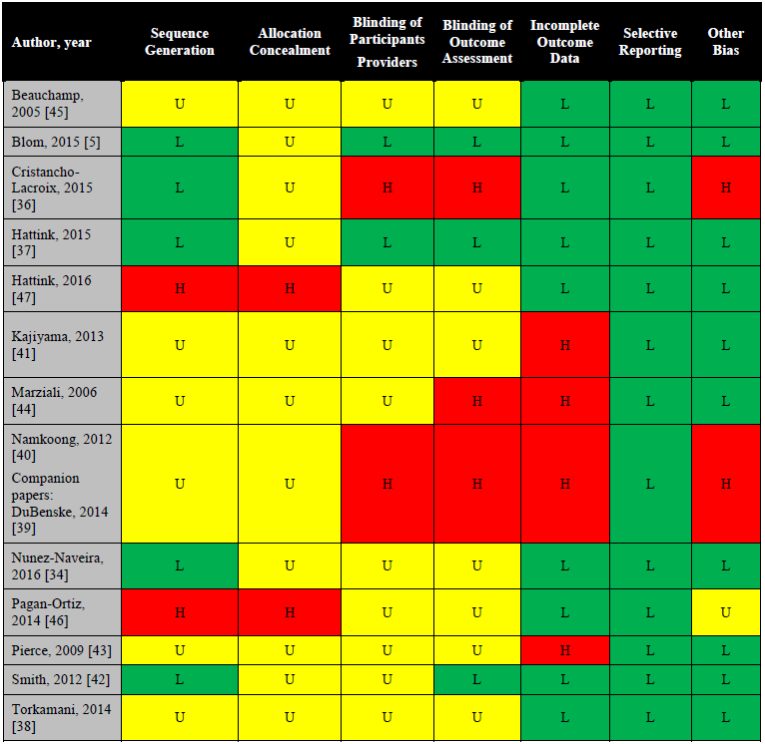
Risk of bias for the included studies. U: unclear bias (yellow); L: low risk of bias (green); H: high risk of bias (red).

The results of the critical appraisal of individual studies for level of bias for sequence generation, allocation concealment, blinding, completeness of outcome assessment, selective reporting, and other biases are shown in Figure 2. Overall, the Cochrane Risk of Bias (RoB) showed mixed quality of study methodology: 2 studies with low RoB [35,37], 3 studies with high RoB [36,40,47], and 8 studies with unclear RoB [34,38,41-46].

### Effectiveness of Internet-Based Interventions

The meta-analysis included an examination of the impact of all internet-based interventions combined as well as an analysis of the impact of each type of internet-based intervention according to mental health outcome. All forest plots are shown in Multimedia Appendix 4.

#### Any Internet-Based Intervention

A summary of the results of the meta-analysis of any internet-based intervention on mental health outcomes is shown in Table 1. Compared to no or minimal internet-based intervention, any type of internet-based intervention resulted in a beneficial mean decrease of 0.48 points (95% CI –0.75 to –0.22) for stress or distress among caregivers and a beneficial mean decrease of 0.40 points (95% CI –0.58 to –0.22) for anxiety among caregivers. There were no statistically significant differences between groups for the mental health outcomes of depression, coping, overall mental health, quality of life, and overall health. Heterogeneity for the combined effect estimate was observed for the mental health outcomes of depression, stress or distress, quality of life, and overall health (*P*<.10) but not for anxiety and coping. The overall GRADE quality of evidence for each outcome ranged from very low to low.

#### Types of Internet-Based Interventions

Mental health outcomes of interest were examined by the different types of internet-based interventions as shown in Table 2. For information or education only interventions, results showed a beneficial mean decrease of 0.31 points (95% CI –0.50 to –0.11) for depression, a beneficial mean decrease of 0.57 points (95% CI –0.77 to –0.37) for stress or distress, and a beneficial mean decrease of 0.42 points (95% CI –0.65 to –0.19) for anxiety among caregivers, compared to minimal or no internet-based intervention. These results were based on moderate quality of evidence. The remaining mental health outcomes of coping, quality of life, and overall health did not show statistically significant differences between groups. Four of the 6 mental health outcomes of interest included only 1 study. No heterogeneity was detected for the mental health outcomes.

For studies that examined information or education plus PPS, there were no differences between intervention and control groups for any of the mental health outcomes in which there were data including depression, stress or distress, quality of life, and overall health. For studies that included information or education plus PFPS as the intervention, results showed a beneficial mean decrease of 0.34 points (95% CI –0.63 to –0.05) for depression and a beneficial mean decrease of 0.36 points (95% CI –0.66 to –0.07) for anxiety among caregivers, compared to minimal or no internet-based intervention. The quality of evidence for each of these outcomes was moderate.

For studies that examined the intervention of information or education plus combined peer and professional psychological support, results showed a beneficial 1.25-point mean increase for overall health (95% CI 0.24 to 2.25) among caregivers, compared to no or minimal internet-based intervention; however, this result was based on 1 study with an overall sample size of less than 20 caregivers and consequently very low quality of evidence. The remaining outcomes showed no differences between groups. There were no differences between groups for the intervention of information or education with telephone monitoring plus combined peer and professional psychological support for the outcome of quality of life. No other mental health outcomes were reported for this type of intervention. See Multimedia Appendix 5 for the full GRADE assessment details.

**Table 1:** Summary of effectiveness of any internet-based interventions.

Mental health outcomes	Number of studies	Intervention/control	Estimate standard mean difference (95% CI)	I^2^ (%)	GRADE^a^ quality assessment
Depression	8	407/422	–0.19 (–0.43 to 0.05)	59^b^	Very low
Stress or distress	6	288/297	–0.48 (–0.75 to –0.22)	49^b^	Low
Anxiety	2	240/239	–0.40 (–0.58 to –0.22)	0	Low
Coping	2	199/204	–0.01 (–0.20 to 0.19)	0	Very low
Overall mental health	1	45/52	–0.29 (–0.69 to 0.11)	—	Very low
Quality of life	4	102/117	0.01 (–0.49 to 0.51)	68^b^	Very low
Overall health	2	34/34	0.35 (–1.30 to 2.00)	88^b^	Very low

^a^GRADE: Grading of Recommendations Assessment, Development, and Evaluation.

^b^Statistically significant heterogeneity (*P*<.10).

**Table 2:** Summary of effectiveness of types of internet-based interventions.

Mental health outcomes		Number of studies		Intervention/control		Estimate standard mean difference (95% CI)		I^2^ (%)		GRADE^a^ quality assessment
**Information or education**										
	Depression		2		196/206		–0.31 (–0.50 to –0.11)		0	Moderate
	Stress/distress		2		196/206		–0.57 (–0.77 to –0.37)		0	Moderate
	Anxiety		1		150/149		–0.42 (–0.65 to –0.19)		—	Moderate
	Coping		1		150/149		0.00 (–0.23 to 0.23)		—	Low
	Overall mental health		1		45/52		–0.29 (–0.69 to 0.11)		—	Very low
	Quality of life		1		46/57		0.33 (–0.06 to 0.72)		—	Very low
	Overall health		1		25/24		–0.44 (–1.01 to 0.13)		—	Very low
**Information or education + PPS^b^**											
	Depression		2		55/55		–0.11 (–0.48 to 0.27)		0	Very low
	Stress/distress		2		52/56		–0.46 (–1.41 to 0.50)		83^c^	Very low
	Quality of life		1		21/25		–0.36 (–0.95 to 0.22)		—	Very low
	Overall health		1		25/24		–0.44 (–1.01 to 0.13)		—	Very low
**Information or education + PFPS^d^**											
	Depression		1		90/90		–0.34 (–0.63 to –0.05)		—	Moderate
	Anxiety		1		90/90		–0.36 (–0.66 to –0.07)		—	Moderate
**Information or education + combined PPS + PFPS**												
	Depression		3		66/71		–0.11 (–1.01 to 0.78)		83^c^	Very low
	Stress/distress		2		40/35		–0.30 (–1.05 to 0.44)		61^c^	Very low
	Coping		1		49/55		–0.03 (–0.41 0.36)		—	Very low
	Overall mental health		1		45/52		–0.29 (–0.69 to 0.11)		—	Very low
	Quality of life		1		18/20		0.55 (–0.10 to 1.20)		—	Very low
	Overall health		1		9/10		1.25 (0.24 to 2.25)		—	Very low
**Information or education + telephone monitoring + combined PPS + PFPS**													
	Quality of life		1		17/15		–0.60 (–1.31 to 0.11)		—	Very low

^a^GRADE: Grading of Recommendations Assessment, Development, and Evaluation.

^b^PPS: peer psychosocial support.

^c^Statistically significant heterogeneity (*P*<.10).

^d^PFPS: professional psychosocial support.

## Discussion

### Principal Findings

Our systematic review and meta-analysis showed small to moderate beneficial effects of internet-based interventions on caregiver mental health including a reduction in symptoms of depression, stress or distress, and anxiety. The types of internet-based interventions that appeared to have a beneficial effect on mental health included information or education only on decreasing depression, stress or distress, and anxiety and information or education plus PFPS on reducing depression and anxiety. Critical appraisal determined a wide range of the quality of evidence but included a moderate quality of evidence for a modest effect size for a beneficial effect among the 2 specific types of internet-based interventions of information or education only and information or education and PFPS. Additional benefits were shown for the internet-based intervention of information or education plus combined peer and psychological support when it came to overall health among caregivers; however, this was based on a small sample size (<20) and a very low quality of evidence.

Accounting for the type of internet-based intervention revealed additional trends not shown when all types of internet-based interventions were combined. The results showed an approximate 20% increase in the magnitude of effect for stress or distress and an information or education only internet-based intervention among 2 studies, compared to when all 6 studies on stress or distress were combined. Symptoms of depression were improved for an information or education only internet-based intervention as well as for an information or education plus PFPS internet-based intervention, not shown when all 8 studies on depression were combined.

Based on the detailed critical appraisal and quality assessment of included studies, there are a number of possible reasons that consistent findings across the mental health outcomes were not shown. According to the GRADE scores, the quality of evidence was poor for a number of the outcomes examined, and none of the outcomes was rated as having high-quality evidence. This may reflect, in part, this new and evolving area of focus and the resulting lack of consistency across studies—for example, not all studies examined the mental health outcomes of interest, there was variability in the measurement tools used to assess the different mental health outcomes, the care recipients across studies differed, and too few studies examined the different types of internet-based interventions resulting in small numbers of studies for some outcomes. No differences were noted for multicomponent internet-based interventions on coping and overall mental health since these outcomes were only examined in a few studies. No differences were noted for quality of life perhaps due to small sample sizes and differences in types of interventions. Studies included in the subgroup analyses by type of internet-based intervention were judged to be predominately of low to very low quality of evidence suggesting a number of methodological limitations. Four studies had high risk of bias in the area of incomplete outcome data; 3 studies had high risk of bias for blinding of outcome assessment; and 2 studies had high risk of bias for sequence generation, allocation concealment, and blinding of participants or providers.

There were also many areas where risk of bias could not be assessed due to lack of information in the published papers. For example, risk of bias related to allocation concealment was rated as unclear in 11 of the 13 interventions assessed. Risk of bias related to blinding of participants and providers was rated as unclear in 9 of the 13 interventions examined. Risk of bias related to blinding of outcome assessors was rated as unclear in 7 of the interventions examined. The provision of more detailed information about trial procedures using the Consolidated Standards of Reporting Trials (CONSORT) guidelines for nonpharmacological interventions [48] would enable more accurate assessments of studies for bias and may over time help to elevate the quality of evidence in this area.

We examined the best studies (those with low risk of bias) [35,37,42] to see if there were further insights to be gained. These studies all demonstrated beneficial effects on mental health outcomes: depression [35,42], anxiety [35], stress [37], and quality of life [37]. However, they included quite different types of internet-based interventions. Blom’s [35] information or education plus PFPS intervention, targeted at caregivers of persons with dementia, included both a Web-based 8-week course and coaching, monitoring, and evaluation provided by a psychologist. Hattink’s [37] information or education plus PPS intervention, also targeted at caregivers of persons with dementia, included a personalized training portal and 2 to 4 months of course materials, interactive exercises, and connection with a Facebook community. Smith’s [42] information or education plus combined peer and professional psychosocial support intervention, targeted at spousal caregivers of stroke survivors, involved an 11-week educational program supported by an experienced cardiovascular nurse manager. These interventions had a lengthier intervention period than some of the other studies, 2 of these studies involved professional support, and 1 involved connections with other caregivers. It is possible that these intervention components hold more promise for improving mental health outcomes of caregivers.

Despite significant findings across a range of evidence quality, the intervention mechanism by which improvements in mental health were achieved is still not clear. The interactivity of the information or education only internet-based interventions may have contributed to our significant findings as previously shown by Guay et al [20]. The previously shown important role of human support [20] was variable in our findings, with a beneficial effect shown for the addition of professional psychological support only. It may be that the needs and experiences of the caregivers targeted in these multicomponent interventions are so diverse that the potential impacts of internet-based interventions are not realized. A theoretical basis for internet-based interventions [20,49] has shown to be impactful, and a number of our included studies reported using theory to develop their interventions [36,42,47]. Many interventions included behavior change techniques such as stress management [34,41], problem solving [35], and graded tasks [37], which may have contributed to significant findings. The most efficacious interventions included caregivers and care recipients who were homogeneous, with caregivers characterized as being mostly older female adults and care recipients being those living with some form of dementia [35-38,41,44,45]. Internet-based interventions, when designed with the target populations in mind, may be more likely to demonstrate a beneficial effect on the mental health of caregivers. Internet-based interventions being developed for caregivers should have a strong theoretical basis [50] and incorporate behavior change techniques, particularly those aimed to help manage stress and enhance coping.

### Strengths and Limitations

This review summarizes the most relevant trial evidence available to assess the benefits of internet-based interventions on caregiver mental health outcomes. All of the available evidence was published between 2005 and 2017, with more literature published recently (from 2013 to 2017), emphasizing the growing interest in internet technology to support caregivers. However, the review identified that the overall quality of evidence ranged from very low to moderate quality. To our knowledge, this is the first systematic review and meta-analysis examining the impact of internet-based interventions on mental health outcomes of caregivers of adults with chronic conditions living in the community. Although this is an emerging field in the literature, our review set out an a priori selection of rigorous methodological designs, including RCTs and CCTs. This systematic review and meta-analysis was completed with a comprehensive search strategy developed to identify relevant and on-topic literature pertaining to internet-based interventions on informal caregiver mental health outcomes. The review was conducted using methodologically rigorous processes for systematic reviews and meta-analyzed the data using appropriate methods for combining studies that used different outcome assessment tools.

The limitations of the review include the methodological weakness of the studies included, despite being RCTs and CCTs. There was considerable heterogeneity in the interventions across studies. Therefore, we analyzed the impact of the internet-based interventions according to the components of the interventions to understand the impact of these components; however, there were too few studies having used each type of internet-based interventions across all of the mental health outcomes of interest.

### Conclusions

This is the first meta-analysis of the impact of internet-based interventions for informal caregivers of adults with chronic conditions on caregiver mental health outcomes. The findings suggest there is an emergence of literature pertaining to internet-based interventions for informal caregivers examining the impact on mental health outcomes. However, future large, high-quality research with clear methodology and consistently reported outcomes of mental health using standardized assessment tools to facilitate meta-analysis and an assessment of clinical relevance are needed to further inform the effectiveness of such interventions, particularly multicomponent internet-based interventions that use peer or professional health care provider support.

## Appendix

Multimedia Appendix 1 Search terms.

Multimedia Appendix 2 Characteristics of included studies.

Multimedia Appendix 3 Mental health outcomes and measurement tools.

Multimedia Appendix 4 Meta-analysis and forest plots.

Multimedia Appendix 5 Detailed Grading of Recommendations, Assessment, Development and Evaluation evidence tables.

## Abbreviations

CCT: controlled clinical trial
CI: confidence interval
CONSORT: Consolidated Standards of Reporting Trials
GRADE: Grading of Recommendations Assessment, Development, and Evaluation
PFPS: professional psychosocial support
PPS: peer psychosocial support
PRISMA: Preferred Reporting Items for Systematic Reviews and Meta-Analyses
RCT: randomized controlled trial
RoB: risk of bias
SE: standard error
SMD: standardized mean difference
