# Supplementary material for: Impact of Internet-Based Interventions on Caregiver Mental Health: Systematic Review and Meta-Analysis
Source: J Med Internet Res. 2018 Jul 3;20(7):e10668. doi: 10.2196/10668 (PMC6053616; doi:10.2196/10668)
Supplement: Multimedia Appendix 1 [file jmir_v20i7e10668_app1.pdf]

Multimedia Appendix 1: Search Terms

Database: Ovid MEDLINE
Date: April 19, 2017
----
1 internet/ or blogging/ or social media/ (60735)
2 Social Networking/ (1672)
3 electronic mail/ or text messaging/ (3328)
4 Telecommunications/ (4555)
5 (internet or web or social media or blog* or social networking or twitter* or tweet* or online or instant* messag* or text messag* or text or texts or texting or facebook* or myspace or linkedin or email* or e-mail* or electronic mail or app or apps or message board* or electronic or e-health or telecommunication*).ti,ab. (357437)
6 webcasts/ (415)
7 (webcast* or podcast* or streaming video* or RSS or really simple syndication or youtube).ti,ab. (2638)
8 or/1-7 (383983)
9 Caregivers/ (25701)
10 Family/ or adult children/ (67923)
11 Spouses/ (8034)
12 (carer* or caretaker* or care-giver* or caregiver*).ti,ab. (54171)
13 (adult child* or spous*).ti,ab. (16966)
14 or/9-13 (141686)
15 exp adult/ (6118268)
16 (adult or adults or senior* or elderly or frail).ti,ab. (1118076)
17 exp Dementia/ (134639)
18 (dementia* or alzheimer*).ti,ab. (157049)
19 or/15-18 (6720735)
20 8 and 14 and 19 (2272)
21 limit 20 to english language (2180)
22 limit 21 to yr="1995 -Current" (2153)
23 remove duplicates from 22 (2044)
****

Database: Embase

----
1 Internet/ (86662)
2 internet.ti,ab. (46608)
3 web.ti,ab. (80187)
4 social media/ (6199)
5 social media.ti,ab. (4199)
6 blog*.ti,ab. (1585)
7 (app or apps).ti,ab. (21008)
8 twitter*.ti,ab. (1501)
9 tweet*.ti,ab. (709)
10 online.ti,ab. (75752)

11 message board*.ti,ab. (173)
12 instant* messag*.ti,ab. (215)
13 text messaging/ (2096)
14 text messag*.ti,ab. (2373)
15 text*.ti,ab. (100263)
16 facebook.ti,ab. (2190)
17 myspace.ti,ab. (102)
18 linkedin.ti,ab. (107)
19 e-mail/ (12518)
20 email*.ti,ab. (10097)
21 e-mail.ti,ab. (8589)
22 webcast/ (236)
23 electronic.ti,ab. (162519)
24 social networking.ti,ab. (1873)
25 (webcast* or podcast* or streaming video* or RSS or really simple syndication or youtube).ti,ab. (3719)
26 or/1-25 (493688)
27 caregiver/ (51625)
28 (carer* or care giver* or caregiver* or caretaker*).ti,ab. (73729)
29 family/ (81236)
30 adult child/ (766)
31 spouse/ (11977)
32 (adult child* or spous*).ti,ab. (20848)
33 or/27-32 (179855)
34 exp adult/ (6168919)
35 pensioner/ (1165)
36 (adult or adults or middle aged or elderly or frail or pensioner*).ti,ab. (1425353)
37 exp Dementia/ (272277)
38 (dementia* or Alzheimer*).ti,ab. (213222)
39 or/34-38 (6927245)
40 26 and 33 and 39 (3077)
41 limit 40 to (english language and yr="1995 -Current") (2947)
****

Database: PsycINFO

----

1 internet/ (25287)
2 social media/ or electronic communication/ or online social networks/ or exp computer mediated communication/ (13808)
3 electronic communication/ or blog/ or text messaging/ (3420)
4 (internet or web or social media or blog* or social networking or twitter* or tweet* or online or instant* messag* or text messag* or text or texts or texting or facebook* or myspace or linkedin or email* or e-mail* or electronic mail or app or apps or message board* or electronic or e-health or telecommunication*).ti,ab. (162560)
5 (webcast* or podcast* or streaming video* or RSS or really simple syndication or youtube).ti,ab. (1350)

6 or/1-5 (166818)
7 caregivers/ (22080)
8 FAMILY/ (35407)
9 adult offspring/ (3677)
10 SPOUSES/ (8003)
11 (carer* or caretaker* or care-giver* or caregiver*).ti,ab. (44136)
12 (adult child* or spous*).ti,ab. (19842)
13 or/7-12 (101151)
14 (adult or adults or senior* or elderly or frail or middle aged).ti,ab. (338325)
15 exp DEMENTIA/ (59133)
16 (dementia* or alzheimer*).ti,ab. (73421)
17 or/14-16 (396607)
18 6 and 13 and 17 (1035)
19 limit 18 to (english language and yr="1995 -Current") (971)
****

Database Name: Cochrane Database

ID Search Hits
#1 MeSH descriptor: [Internet] explode all trees 2567
#2 MeSH descriptor: [Social Networking] this term only 38
#3 MeSH descriptor: [Electronic Mail] explode all trees 241
#4 MeSH descriptor: [Text Messaging] explode all trees 281
#5 MeSH descriptor: [Telecommunications] this term only 92
#6 (internet or web or social media or blog* or social networking or twitter* or tweet* or online or instant* messag* or text messag* or text or texts or texting or facebook* or myspace or linkedin or email* or e-mail* or electronic mail or app or apps or message board* or electronic or e-health or telecommunication*):ti,ab,kw (Word variations have been searched) 21220
#7 MeSH descriptor: [Webcasts] this term only 0
#8 (webcast* or podcast* or streaming video* or RSS or really simple syndication or youtube):ti,ab,kw (Word variations have been searched) 210
#9 S #1 or S #2 or S #3 or S #4 or S #5 or S #6 or S #7 or S #8 14057
#10 MeSH descriptor: [Caregivers] this term only 1485
#11 MeSH descriptor: [Family] this term only 1163
#12 MeSH descriptor: [Adult Children] this term only 19
#13 MeSH descriptor: [Spouses] this term only 257
#14 (carer* or caretaker* or care-giver* or caregiver*):ti,ab,kw (Word variations have been searched) 5497
#15 (adult child* or spous*):ti,ab,kw (Word variations have been searched) 27496
#16 s #10 or s #11 or s #12 or s #13 or S #14 or S #15 22270
#17 MeSH descriptor: [Adult] explode all trees 1574
#18 (adult or adults or senior* or elderly or frail):ti,ab,kw (Word variations have been searched) 417305
#19 MeSH descriptor: [Dementia] explode all trees 4287
#20 (dementia* or alzheimer*):ti,ab,kw (Word variations have been searched) 9936
#21 s #17 or s #18 or S #19 or S #20 261413

#22 s #9 and s #16 and s #21 Publication Year from 1995 to 2017 940

Database CINAHL

Query Limiters/Expanders Last Run Via Results
S25 S11 AND S17 AND S22 Limiters Published Date: 1995010120161231; English Language
S24 S11 AND S17 AND S22 Limiters Published Date: 1995010120170419
S23 S11 AND S17 AND S22 Search modes Boolean/Phrase
S22 S18 OR S19 OR S20 OR S21
S21 TI ((dementia* or alzheimer*)) OR AB ((dementia* or alzheimer*))
S20 (MH "Dementia+")
S19 TI ((adult or adults or senior* or elderly or frail or pensioner* or middle aged)) OR AB ((adult or adults or senior* or elderly or frail or pensioner* or middle aged))
S18 (MH "Adult+")
S17 S12 OR S13 OR S14 OR S15 OR S16
S16 TI ((adult child* or spous*)) OR AB ((adult child* or spous*))
S15 (MH "Spouses")
S14 (MH "Family") OR (MH "Adult Children")
S13 TI ((carer* or caretaker* or caregiver* or caregiver*)) OR AB ((carer* or caretaker* or caregiver* or caregiver*))
S12 (MH "Caregivers")
S11 OR S5 OR S6 OR S7 OR S8 OR S9 OR S10
S10 (MH "Social Networking")
S9 (MH "Webcasts+")
S8 TI ((webcast* or podcast* or streaming video* or RSS or really simple syndication or youtube)) OR AB ((webcast* or podcast* or streaming video* or RSS or really simple syndication or youtube))
S7 TI ((internet or web or social media or blog* or social networking or twitter* or tweet* or online or instant* messag* or text messag* or text or texts or texting or facebook* or myspace or linkedin or email* or email* or electronic mail or app or apps or message board* or electronic or ehealth or telecommunication*)) OR AB ((internet or web or social media or blog* or social networking or twitter* or tweet* or online or instant* messag* or text messag* or text or texts or texting or facebook* or myspace or linkedin or email* or email* or electronic mail or app or apps or message board* or electronic or ehealth or telecommunication*))
S6 (MH "Social Media")
S5 (MH "Electronic Mail")
S4 (MH "Blogs")
S3 (MH "World Wide Web Applications")

S2 (MH "World Wide Web")
S1 (MH "Internet") Search modes Boolean/Phrase
Interface EBSCOhost Research Databases Search Screen Advanced Search

Database Name: Ageline

S1 ((DE "Internet") OR (AB (internet OR web OR social media OR blog* OR social networking OR twitter* OR tweet* OR online OR instant* messag* OR text messag* OR text OR texts OR texting OR facebook* OR myspace OR linkedin OR email* OR e-mail* OR electronic mail OR app OR apps OR message board* OR electronic OR e-health OR telecommunication*)) OR (AB (webcast* OR podcast* OR streaming video* OR RSS OR really simple syndication OR youtube))) AND ((DE "Caregivers") OR (DE "Adult Children" OR DE "Spouses") OR (AB (carer* OR caretaker* OR care-giver* OR caregiver*)) OR (AB (adult child* OR spous*))) AND ((DE "Adults of All Ages") OR (AB (adult OR adults OR senior* OR elderly OR frail)) OR (DE "Dementia" OR DE "Alzheimers Disease" OR DE "Early Onset Dementia" OR DE "Frontotemporal Dementia" OR DE "Lewy Body Dementia" OR DE "Vascular Dementia") OR (AB (dementia* OR

alzheimer*)))
Results 3,082
