# Supplementary material for: Impact of Internet-Based Interventions on Caregiver Mental Health: Systematic Review and Meta-Analysis
Source: J Med Internet Res. 2018 Jul 3;20(7):e10668. doi: 10.2196/10668 (PMC6053616; doi:10.2196/10668)
Supplement: Multimedia Appendix 2 [file jmir_v20i7e10668_app2.pdf]

Multimedia Appendix 2: Detailed Characteristics of Included Studies
Risk of Bias: Low (L); High (H); Unclear (U)

**Study/Location**	**Beauchamp, 2005, US [48]**
**Purpose**	Our goal was to evaluate the efficacy of a multimedia support program delivered over the Internet to employed family caregivers of persons with dementia
**Methods**	Design: RCT<br>Setting/ Recruitment Methods: We recruited participants through a national campaign that included wire service advertising, contact notices on corporate Web sites, e-mail announcements on caregiver-related listserves, newsletter articles, and worksite promotional flyers. Interested persons were directed to a Web site that described the study and provided an online screening survey.<br>Inclusion criteria: To allow for the broadest representation of caregivers in the workforce, we required participants to be employed at least part time and have at least four contacts a month caring for a family member with substantial memory problems.
**Participants**	Recruited Sample: 299<br>Baseline Sample: I = 150; C = 149<br>Loss to follow-up: 8 overall (307 participated through the 30-day follow-up assessment, 299 had complete data on all measures)<br>Mean age (years): Overall = 46.9 (12.2)<br>Gender [Male n (%)]: 27%<br>Relationship of caregiver to care recipient: cared for a parent (67%), a spouse or partner (7%), some other relative (23%), or a nonrelative (3%)<br>Ethnicity: Eighty percent of evaluation participants were Caucasian, 4% African American, 8% Hispanic, and 8% other<br>SES status: Ninety percent had completed at least some college or trade school

**Intervention**	Description of Intervention: Caregiver’s Friend: Dealing With Dementia is a Web-based multimedia intervention that provides text material and videos that model positive caregiving strategies. Funded by the National Institute on Aging, program content was created for this project by research scientists and instructional designers on the basis of an extensive literature review; interviews with academic gerontologists, social workers, nurses; and focus groups with an ethnically and geographically diverse range of family caregivers.<br><br>Description of Control: A usual care waitlist control group with no attention-placebo. We informed participants in the control condition by e-mail that they would receive an e-mail in 30 days inviting them to answer another set of questions (the 30-day, T2 follow-up), at which time they would be free to view Caregiver’s Friend.<br><br>Duration of Intervention: 30 days<br><br>Length of follow-up: 30 days
**Risk of Bias**<br><br>**Overall: U**	Selection: (U) Insufficient information to make assessment<br><br>Allocation Concealment: (U) Insufficient information to make assessment<br><br>Blinding Participants: (U) Insufficient information to make assessment<br><br>Blinding Assessors: (U) Insufficient information to make assessment<br><br>Incomplete Data: (L) Drop-out rates =<10%<br><br>Selective Reporting: (L) Outcomes in methods and results<br><br>Other: (L) No baseline imbalance

**Study/Location**	**Blom, 2015, The Netherlands [38]**
**Purpose**	To study the effectiveness of an innovative guided self-help Internet course "Mastery over Dementia" which is designed to reduce caregivers' symptoms of depression and anxiety.
**Methods**	Design: RCT<br><br>Setting/Recruitment Methods: From 1 April 2010 until 31 December 2011, family caregivers of people with dementia were recruited via the website "Mastery over Dementia" the monthly digital newsletter of the Alzheimer's Society, leaflets at Alzheimer's Cafe meetings (meetings for people with dementia, their caregivers, and other interested people) and information letters to memory clinics and other relevant care institutes. After expressing interest in participating, caregivers were sent an

	information letter with more details of the study. There were asked to complete and sign a written informed consent form and return it by mail.<br>Inclusion criteria: Caregivers who had at least some symptoms of depression or anxiety or feelings of burden were included.
**Participants**	Recruited Sample: 245<br>Baseline Sample: I = 149; C = 96<br>Loss to follow-up: 59 in intervention group, 11 in comparison group<br>Mean age (years): Overall = 61.2<br>Gender [Male n (%)]: 30.6%<br>Relationship of caregiver to care recipient: Spouses (58.4%); children (in Law) (39.6%)<br>Ethnicity: Dutch nationality (99.2%)<br>SES status: The level of education varied from primary school to university, with 47.3% holding a bachelor's degree or higher.
**Intervention**	Description of Intervention: Experimental group: Internet course Mastery over Dementia (MoD). The Internet course consists of 8 lessons and a booster session with the guidance of a coach monitoring the progress of participants and evaluating the homework. Each lesson has the same structure and consists of information (text material and videos), exercises, and homework, with an evaluation at the start and end of each session. The elements of the course are presented in the following order: coping with behavioral problems (problem solving); relaxation; arranging help from others; changing non-helping thoughts into helping thoughts (cognitive restructuring); and communication with others (assertiveness training).<br>Description of Control: Caregivers in the comparison group received a minimal intervention consisting of e-bulletins (digital newsletters) with practical information on providing care for someone with dementia. The bulletins were sent by email according to a fixed schedule (every 3 weeks) over nearly 6 months. The topics of the bulletin, which did not overlap with the content of MoD, were driving; holiday breaks; medication; legal affairs; activities throughout the day; help with daily routines; grieving; safety measures in the home; and possibilities for peer support. There was no contact with a coach.<br>Duration of Intervention: 5-6 months<br>Length of follow-up: Last assessment 5-6 months after baseline.

**Risk of Bias Overall: (L)**	Selection: (L) computerized random-number/block randomization<br>Allocation Concealment: (U) Insufficient Information for assessment<br>Blinding Participants: (L) No concerns<br>Blinding Assessors: (L) No concerns<br>Incomplete Data: (L) Intention to Treat (ITT) Analysis<br>Selective Reporting: (L) Outcomes in methods and results<br>Other: (L) No concerns

**Study/Location**	**Cristancho-Lacroix, 2015, France [39]**
**Purpose**	This study aimed to evaluate through a pilot unblinded randomized controlled trial the efficacy and acceptability of a Web-based psychoeducational program for informal caregivers of persons with Alzheimer's disease (PWAD) based on a mixed methods research design
**Methods**	Design: RCT<br>Setting/Recruitment Methods: The recruitment strategy included flyers and posters placed in the hospital. During the consultations, geriatricians proposed this protocol to caregivers of PWAD. The caregivers interested in the study filled out a contact form. Then a psychologist provided them with the information form, confirmed inclusion criteria, and collected the signed informed consent.<br>Inclusion criteria: Eligible participants were required to be French-speaking caregivers of community-dwelling PWAD who met the criteria of the Diagnostic and Statistical Manual of Mental Disorders, 4th Edition. Caregivers had to spend at least 4 hours per week with their relative, be aged 18 years or older, scored 12 or more on the Perceived Stress Scale (PSS-14), and to have access to a computer with Internet connection. Professional caregivers were ineligible.
**Participants**	Recruited Sample: 49<br>Baseline Sample: I = 25; C = 24<br>Loss to follow-up: (I = 3; C = 3)<br>Mean age (years): Overall, I = 64.2 (10.3); C = 59.0 (12.4)<br>Gender [Male n (%)]: I = 36%; C = 33%<br>Relationship of caregiver to care recipient: Children (I=64%, C=67%) and spouses

	Ethnicity: NR<br>SES status: High Level of Education I=76%, C=75%, Middle level of education I=24% C=12%.
**Intervention**	Description of Intervention: The experimental group participants received at baseline a 10-minute training session on how to use the website, a log-in and password, a printed version of the user's manual, and a notebook to write personal ideas about their application of the program's content. Each week, participants had to read through an entire thematic session and fill out a printed satisfaction questionnaire. Other website sections (e.g., relaxation training, forum) were available but not mandatory to complete the program. No modification regarding methodology, program content (except for forum discussions), or the website was done during the course of the study.<br>Description of Control: The control and experimental group participants received usual care, in which they were provided with information about the illness during their semiannual follow-up with their geriatrician. The control group participants were given access to the Diapason program at the end of their participation. All participants were advised to look for additional help if necessary and were asked to inform the researcher about it.<br>Duration of Intervention: 3 months (12 weekly online sessions)<br>Length of follow-up: 3 months post intervention.
**Risk of Bias**<br>**Overall: (H)**	Selection: (L) Computerized random-number/block randomization<br>Allocation Concealment: (U) Insufficient information for assessment<br>Blinding Participants: (H) No blinding<br>Blinding Assessors: (H) No blinding<br>Incomplete Data: (L) No concerns<br>Selective Reporting: (L) No concerns<br>Other: (H) Baseline imbalance between groups

**Study/Location**	**Hattink, 2015, the Netherlands [40]**
**Purpose**	The objective of the current study was to evaluate the user friendliness, usefulness, and impact of STAR with informal caregivers, volunteers and professional caregivers

**Methods**	Design: RCT<br>Setting/Recruitment Methods: Participants in the Netherlands were recruited through meeting centers for people with dementia and their caregivers, regional branches of the national Alzheimer's organizations, case managers, care organizations, and via announcements through several informative websites targeted at informal caregivers, volunteers, and those with an interest in dementia. In the United Kingdom, participants were recruited through caregivers' cafes, church groups, university service users and caregiver groups, and local dementia care and welfare organization<br>Inclusion criteria: Participants were caring for someone with dementia as an informal caregiver, a volunteer in dementia care, or a professional caregiver, and were living in either the Netherlands or in the United Kingdom
**Participants**	Recruited Sample: 142<br>Baseline Sample: I = 27; C = 32 (informal caregivers)<br>Loss to follow-up: NR<br>Mean age (years): Overall, I = 52.9 (11.43); C = 54.69 (14.36)<br>Gender [Male n (%)]: I = 26.0%; C = 31.0%<br>Relationship of caregiver to care recipient: Partner; child; sibling<br>Ethnicity: NR<br>SES status: NR
**Intervention**	Description of Intervention: The STAR platform was designed to be accessible through any Internet-enabled device so users could access the course at any time and place of their convenience. The STAR training portal consists of an online course with 8 modules relating to different topics. These topics were selected to cover a wide range of topics relating to dementia and dementia care. The modules consist of text, videos, interactive exercises, knowledge tests, and also include references to other websites, literature, and videos<br>Description of Control: People in the control group were informed that they were assigned to the group that could follow the course free of charge after post-test measurements 4 months later<br>Duration of Intervention: 2-4 months<br>Length of follow-up: immediate post (2-4 months)
**Risk of Bias**<br>**Overall: (L)**	Selection: (L) Computer Generated<br>Allocation Concealment: (U) Insufficient information for assessment

	Blinding Participants: (L) No concerns<br>Blinding Assessors: (L) No concerns<br>Incomplete Data: (L) No concerns<br>Selective Reporting: (L) No concerns<br>Other: (L) No concerns

**Study/Location**	**Hattink, 2016, the Netherlands, Germany and Belgium [50]**
**Purpose**	This research aimed to integrate three previously developed assistive technology (AT) systems into one modular, multifunctional system, which can support people with dementia carers throughout the course of dementia
**Methods**	Design: RCT (Germany) and pre-test–post-test control group design with matched groups (Netherlands and Belgium)<br>Setting/Recruitment Methods: Care organizations in the three countries via letters (additional information meeting in Germany). Those who consented to participated where then contacted for in-home baseline assessments.<br>Inclusion criteria: participants with MCI or dementia living in the community and their informal carers. Other general inclusion characteristics included a maximum of seven rooms in a one story-house with a maximum size of 180 square meters and a maximum of five exterior doors. The house had to have the possibility for installation of wireless broad-band internet access (if not available already), since all signals of the Rosetta system were transmitted wirelessly and uploaded through an internet server.
**Participants**	Recruited Sample: 42 persons with either mild cognitive impairment (MCI) or dementia (19 in the Netherlands, 11 with MCI in Germany and 12 in Belgium), and 32 of their informal carers.<br>Baseline Sample: Intervention = 5 in Germany 15 (combined) in Netherlands and Belgium; Control = 6 in Germany, 16 (combined) in Netherlands and Belgium<br>Loss to follow-up: Including drop out: I=9 and C = 9.<br>Mean age (years): Overall, I = 66; C = 69<br>Gender [Male n (%)]: I = 7/17 (41.2%), C = 6/15 (40.0%)<br>Relationship of caregiver to care recipient: Most carers were either partners or children of the person with dementia.

	Ethnicity: NR<br>SES status: NR
**Intervention**	Description of Intervention: The Rosetta system was installed in the homes of the persons with MCI or dementia in the experimental group. After installation, participants received a training explaining how the system works. The effective usage period varied from half a month (which was the case for one participant, recruited as replacement for a drop-out) to eight months. Average use was nearly four months<br>Description of Control: Persons in the control group received care and support as usual. This usual care generally consisted of home care for household chores and/or personal care and day care. Some participants received extra care, for example, help with food preparation or visits to the general practitioner.<br>Duration of Intervention: 2 weeks to 8 months<br>Length of follow-up: NR
**Risk of Bias**<br>**Overall: (H)**	Selection: (H) Initially designed as RCT for all three regions (Germany, Netherlands, Belgium), however RCT only maintained in Germany.<br>Allocation Concealment: (H) Pre/post-test control group design with matched groups.<br>Blinding Participants: (U) Insufficient information for assessment<br>Blinding Assessors: (U) Insufficient information for assessment<br>Incomplete Data: (L) No concerns<br>Selective Reporting: (L) No concerns<br>Other: (L) No concerns

**Study/Location**	**Kajiyama, 2013, US [44]**
**Purpose**	Determine if the online iCare Stress Management e-Training Program reduces stress, bother, depression, and poor life quality for dementia family caregivers (CGs)
**Methods**	Design: RCT<br>Setting/ Recruitment Methods: CGs were recruited from the community through notices placed in family service agencies and other information and referral resources. Interested CGs contacted research personnel at Photozig, Inc via email or telephone. Following the initial contact all

	communications with interested persons occurred online using questionnaires.<br><br>Inclusion criteria: Screening was a two-step process. In the initial screening, we asked if: 1) they were at least 21 years of age or older; 2) they were caring for an individual with a clinical diagnosis of some type of dementia; and 3) they had access to the Internet on any type of computer or had access to a DVD player. If they answered 'yes' to these questions, they were asked to read the consent form and return a signed copy (either e-mail or regular mail) to indicate willingness to be in the study.
**Participants**	Recruited Sample: 150<br><br>Baseline Sample: I = 75; C = 75<br><br>Loss to follow-up: I = 29; C = 18<br><br>Mean age (years): Overall = NR, I = 55.22 (11.31); C = 57.02 (12.53)<br><br>Gender [Male n (%)]: I = 8 (17%); C = 8 (14%)<br><br>Relationship of caregiver to care recipient: spouse/partner: I = 26 (56%), C = 29 (51%); child I = 15 (33%); C = 21 (37%); other relative: I = 2 (4%); C= 4 (7%); non-relative I = 3 (7%); C = 3 (5%)<br><br>Ethnicity: Caucasian; I = 41 (89%); C = 48 (84%)<br><br>SES status: High school Ed: I=7(15) C=12(21). College: I=26 (57) C=26(46). Graduate: I=13(28); C=19(33)
**Intervention**	Description of Intervention: iCare (ICC)<br><br>In the first year of this project, extensive interviews were conducted with caregivers and professionals in the field to develop and test each of the six modules included in the final web-based program. Table 3 provides a brief outline of the Introduction, the modules, and the summary of future actions included in the intervention. The format for completing the ICC was configured so that the modules had to be completed in the order listed in Table 3. There were no minimum time constraints for completing a module built into the program, but participants were encouraged to practice specific assignments in each module over a 7- to 10-day interval before moving to the next one. The iCare program begins with an information segment about what 'dementia' means and what are common problems associated with it. Then, there are components on dealing with stress including techniques for relaxation, stress management, and challenging unduly negative thoughts about caregiving; behavioral activation (increasing everyday positive activities for oneself and the PWD); communication skills to improve help-seeking with family and community institutions as well as improving ability to relate to the PWD; managing difficult behaviors of the PWD; and finally, a review of

	‘healthy habits’ (nutrition and exercise) for the CG along with information on national resources they can consult for further on-going assistance.<br>Description of Control: CGs assigned to the EOC were exposed to a website containing the similar navigational features, but the content focused on information about dementia, obtained from reputable national sources such as the ADEAR program of the National Institute on Aging and the national website of the Alzheimer’s Association. In addition, links to certain video-taped information were provided (e.g., the Alzheimer’s Project, developed by HBO in collaboration with the National Institute on Aging and the Alzheimer’s Association). Written materials from various health agencies were also provided in a booklet format.<br>Duration of Intervention: 3 months.<br>Length of follow-up: No additional follow ups were completed.
**Risk of Bias**<br>**Overall: (U)**	Selection: (U) Insufficient information for assessment<br>Allocation Concealment: (U) Insufficient information for assessment<br>Blinding Participants: (U) Insufficient information for assessment<br>Blinding Assessors: (U) Insufficient information for assessment<br>Incomplete Data: (H) High attrition with only completers analyses<br>Selective Reporting: (L) No concerns<br>Other: (L) No concerns

**Study/Location**	**Marziali, 2006, Canada [47]**
**Purpose**	The aim of this pilot feasibility study was to evaluate the effects of an innovative, Internet-based psychosocial intervention for family caregivers of older adults with neurodegenerative disease.
**Methods**	Design: RCT<br>Setting/Recruitment Methods: The sampling strategy focused on identifying 66 caregivers of relatives with moderate level disability distributed across three forms of neurodegenerative disease: Alzheimer’s, stroke related dementia, and Parkinson’s. Geriatricians at two participating hospitals located in two remote areas in Canada (the project sites) used their own clinical judgments of moderate-level disability at time of referral. However, time constraints imposed by the funding agencies truncated the recruitment phase, resulting in the need to accept referrals regardless of disability level.

	Inclusion criteria: Geriatricians at two participating hospitals located in two remote areas in Canada (the project sites) used their own clinical judgments of moderate-level disability at time of referral.
**Participants**	Recruited Sample: 66<br>Baseline Sample: I = 33; C = 33 (22 caregivers per disease-specific cohort)<br>Loss to follow-up: I = 10; C = 18<br>Mean age (years): Overall = 67.8<br>Gender [Male n (%)]: 24%<br>Relationship of caregiver to care recipient: authors only indicate that caregivers were relatives of the care recipient.<br>Ethnicity: NR<br>SES status: More than half had annual income of less than $40 000
**Intervention**	Description of Intervention: Technicians at each site installed equipment in the participants' homes and provided two computer training sessions using the Caring for Others computer training manual. Research assistants (not blind to group) administered questionnaires to caregiver participants in their homes at bseline and 6-month follow-up. Questionnaires included (a) the Health Status Questionnaire 12 , an abbreviated version of the validated Medical Outcomes Study 36 ; (b) the Center for Epidemiologic Studies–Depression scale , a short self-report of depressive affect and behavior that is used extensively in medical and mental health studies; (c) a measure that required the caregiver to endorse the presence or absence of activities of daily living (ADLs) and instrumental ADLs (IADLs) performed on behalf of the care recipient (in the current study, for each endorsed ADL and IADL, we asked participants to rate the degree of stress experienced on a 3-point severity scale); (d) the Revised Memory and Behavior Problems Checklist , which asks the caregiver to rate levels of distress experienced in relation to managing the patient's difficult and/or disruptive behaviors; and (e) the Multidimensional Scale of Perceived Social Support a 12-item scale that measures the respondent's perceptions of the availability of social support. All measures are those typically used in studies of dementia caregiver responses to intervention programs.<br>Description of Control: No intervention

	Duration of Intervention: 22 weeks<br>Length of follow-up: 6 months
**Risk of Bias**<br>**Overall: (U)**	Selection: (U) Insufficient information for assessment<br>Allocation Concealment: (U) Insufficient information for assessment<br>Blinding Participants: (U) Insufficient information for assessment<br>Blinding Assessors: (H) Research assistants not blinded administered questionnaires to caregiver participants<br>Incomplete Data: (H) 54% dropout of control participants<br>Selective Reporting: (L) No concerns<br>Other: (L) No concerns

**Study/Location**	**DuBenske, 2014 [42]**, **Namkoong, US, 2012 [43], Gustafson, 2013, USA [51]**
**Purpose**	In this study, the authors examined the effectiveness of an online support system (Comprehensive Health Enhancement Support System) versus the internet in relieving physical symptom distress in patients with non–small cell lung cancer (NSCLC)
**Methods**	Design: RCT<br>Setting/ Recruitment Methods: Dyads were identified by their oncologist and were invited to participate by the enrollment coordinator at each site.<br>Inclusion criteria: Eligible dyads consisted of English-speaking adult patients with NSCLC at stage IIIA, IIIB, or IV and a patient-identified primary caregiver who was willing to participate in the study. Patients had to have a clinician-perceived life expectancy of at least 4 months; if patients had brain metastasis, then it had to be stable. Caregivers provided instrumental, emotional, and/or financial support for the patient and were aged at least 18 years.
**Participants**	Recruited Sample: 285<br>Baseline Sample: I = 144; C = 141<br>Loss to follow-up: I = 49; C = 51<br>Mean age (years): Overall, I = 54.57 (12.21); C = 56.56 (12.86)

	Gender [Male n (%)]: I = 29.5%; C = 33.9%<br>Relationship of caregiver to care recipient: spouse/partner 72%<br>Ethnicity: NR<br>SES status: Highest level of education high school or less: I = 24 (19.9%). C = 27 (22.5%); Some college courses: I = 28 (23.1); C= 34(28.3); Advanced degree: I = = 69 (57); C=59 (49.2)
**Intervention**	Description of Intervention: Those in the CHESS arm received password-protected access to the CHESS Coping with Lung Cancer website. CHESS was designed to be easy to use and: 1) to provide well organized lung cancer, caregiving, and bereavement information; 2) to serve as a channel for communication with and support from peers, experts, clinicians, and users' social networks; 3) to act as a coach by gathering information from users and providing feedback based on algorithms (decision rules); and 4) to provide tools (e.g., a program to organize support from family and friends) to improve the caregiving experience. CHESS included a clinician report that summarized caregiver and patient ratings of the patient's health status and listed their questions for the next clinic visit. Clinicians received e-mail alerts before a scheduled visit and whenever a symptom rating exceeded 7 on a scale from 0 to 10. CHESS services are described in more detail elsewhere.<br>Description of Control: Participants in the Internet arm received training on using the Internet and a list of Internet sites about lung cancer<br>Duration of Intervention: 6 months (but access for up to 2 years)<br>Length of follow-up: 6 months
**Risk of Bias**<br>**Overall: (H)**	Selection: (U) participants are assigned by random numbers but authors do not indicate if the sequence was computer generated<br>Allocation Concealment: (U) Insufficient information for assessment<br>Blinding Participants: (H) Study non-blinded<br>Blinding Assessors: (H) Study non-blinded<br>Incomplete Data: (H) High attrition<br>Selective Reporting: (L) No concerns<br>Other: (H) Baseline imbalance

**Study/Location**	**Nunez-Naveira, 2016, Denmark, Poland, Spain [37]**
**Purpose**	To test an e-learning platform for informal caregivers to explore the

	technical and pedagogical specifications, as well as evaluating the impact of its use on the psychological status of the participants.
**Methods**	Design: RCT<br>Setting/Recruitment Methods: Participants were recruited again from different local Alzheimer's associations of adult day-care centers: from the Danish Alzheimer Association (DAA) and the Skanderborg municipality (SKAN) in Denmark, from Poznan, Walcz, Ciechocinek, and Koszalin in Poland, and from the Gerontological Complex La Milagrosa, Saraiva-Mar´ın, and Afal-Ferrolterra in Spain<br>Inclusion criteria: The inclusion criteria for the study were as follows: (1) taking care of a person diagnosed with dementia by a specialist or a neurologist, according to the criteria of the Classification of Mental and Behavioural Disorders, 10th revision, or the Diagnostic and Statistical Manual of Mental Disorders, 4th edition, text revision , or the National Institute of Neurological Disorders and Stroke-Alzheimer Disease and Related Disorders Association; (2) being the primary caregiver in the following aspects: executing basic care tasks for a minimum of 6 weeks, receiving no remuneration for caregiving service (except from a few Danish caregivers receiving economical compensation for reducing their ordinary working hours while caring for their demented relative), and devoting much time to patient care; (3) suffering a burden according to the 22-item version of the Zarit Burden Interview, using a cut-off point of 24, which was determined to identify family caregivers who are at risk for depression; and (4) signing the informed consent form to participate in the study. The exclusion criteria were to present some of the following conditions that might prevent the evaluation of the participant or the interaction with the platform: cognitive impairment, illiterate, severe hearing and visual or motoric problems.
**Participants**	Recruited Sample: 77<br>Baseline Sample: I = 36; C = 41<br>Loss to follow-up: I = 6; C = 10<br>Mean age (years): NR<br>Gender [Male n (%)]: I = 9 (30.0%); C = 13 (41.9%)<br>Relationship of caregiver to care recipient: The most frequent forms of support used were relative and friends (44.3%)<br>Ethnicity: NR<br>SES status: 46.5% medium level of education

**Intervention**	Description of Intervention: The understAID consists on a Learning section with a database of contents organized in 5 modules with information about 15 different topics. The topics cover information about the care of a person with dementia and caring for oneself as a caregiver. The topics consist of text, videos, and images and they also include references to other websites. The modules and topics included in understAID are Module 1, Cognitive Declines (Topics: Attention, Memory, and Orientation); Module 2, Daily Tasks (Topics: Bathing, Incontinence, Massage and Touch, and Physical Exercises); Module 3, Behavioural Changes (Topics: Anxiety and Agitated Behaviour, Depressive Mood, Manic Symptoms, and Emotional Control and Recognition); Module 4, Social Activities (Communication and Apathy and Loss of Motivation); and Module 5, You as a Caregiver (Topics: Coping with Own Stress and Motivation). It also contains a Daily Task section with the option of using a calendar and reminders for appointments and medication intake. Additionally, it has a Social Network section where the caregivers can interact with other participants and exchange information and opinions. This section was moderated by the researchers of the study.<br><br>Description of Control: Participants in the control group did not use the application and maintained their usual lifestyle<br><br>Duration of Intervention: 3 months<br><br>Length of follow-up: immediate post (3 months)
**Risk of Bias**<br><br>**Overall: (U)**	Selection: (L) Computer generated<br><br>Allocation Concealment: (U) Insufficient information for assessment<br><br>Blinding Participants: (U) Insufficient information for assessment<br><br>Blinding Assessors: (U) Insufficient information for assessment<br><br>Incomplete Data: (L) No concerns<br><br>Selective Reporting: (L) No concerns<br><br>Other: (L) No concerns

**Study/Location**	**Pagan-Ortiz, 2014, USA [49]**
**Purpose**	To assess the effectiveness of a website developed to increase knowledge of ADRD, caregivers' self-efficacy for caregiving (competence), perceived social support, and decreasing caregiver burden and emotional distress

**Methods**	Design: CCT<br>Setting/Recruitment Methods: “Participants were "recruited from an English as a Second Language class at a community-based organization in Boston, Massachusetts. Participants from Puerto Rico and Massachusetts were recruited via outreach strategies that included: letters, press releases, flyers, as well as phone calls to agencies in contact with caregivers. In Mexico, participants were recruited from a pool of caregivers who received social support services at a neurology teaching hospital.<br>Inclusion criteria: NR
**Participants**	Recruited Sample: 72<br>Baseline Sample: I = 17; C = 23<br>Loss to follow-up: I = 2; C = 6<br>Mean age (years): Overall, Range 42 to 78<br>Gender [Male n (%)]: NR<br>Relationship of caregiver to care recipient: NR<br>Ethnicity: 100% Spanish speaking<br>SES status: NR
**Intervention**	Description of Intervention: The intervention group went through four sessions of approximately 1 to 1-1/2 hr each. The first intervention group session (pretest session) was devoted to providing an overview of the study, familiarizing the caregiver with Cuidate Cuidador, and administering the pretest. The next two intervention group sessions were devoted to ensuring participants’ ability to use Cuidate Cuidador’s key features. Caregivers who were not computer-literate received specific assistance to navigate through the web site’s key component features. The fourth session (posttest session) took place at the 1-month point, and was devoted to the administration of the posttest evaluation, as well as a general debriefing for the study.<br>Description of Control: Participants assigned to the control group completed two sessions. A first session was devoted to providing an overview of the study, and administering the pretest. Participants received printed Spanish-language educational materials on Alzheimer’s caregiving. The content covered in the printed materials were similar to the topics offered in Cuidate Cuidador, but were obtained from other sources. Participants were instructed to take time between the pretest and posttest to review and use the educational materials as a reference. The second session (posttest session) took place at a 1-month follow-up, and was devoted to the administration of the post-test outcome measures, as well as a debriefing.

	Duration of Intervention: 1 month<br>Length of follow-up: immediate post (1 month)
**Risk of Bias Overall: (H)**	Selection: (H) Non-randomized<br>Allocation Concealment: (H) Non-randomized<br>Blinding Participants: (U) Insufficient information for assessment<br>Blinding Assessors: (U) Insufficient information for assessment<br>Incomplete Data: (L) No concerns<br>Selective Reporting: (L) No concerns<br>Other: (U) Insufficient information for assessment

**Study/Location**	**Pierce, 2009, USA [46]**
**Purpose**	We hypothesized that carers of stroke survivors who participate in the Web-based intervention, Caring Web would have higher well-being than non-Web users. We also postulated that those survivors whose carers participated in Caring Web would use fewer healthcare services.
**Methods**	Design: RCT<br>Setting/ Recruitment Methods: Subjects were recruited from four rehabilitation centres from which first-time stroke survivors were discharged to home in two Midwestern states.<br>Inclusion criteria: For this study, subjects were the primary person (4age 21) responsible for providing day-to-day care for a person with a first-time stroke who had completed treatment and was discharged to home in northern Ohio or southern Michigan. In addition, carers were required to read, write and understand English and have a telephone and television to facilitate MSN TV and Internet access. All potential subjects were novice Internet users. Subjects assigned to either the Web or non-Web user group did not have Internet access in their homes at the time of enrolment into the study. Non-Web users were also told not to purchase or use Internet service during the study but received usual medical care.
**Participants**	Recruited Sample: 144<br>Baseline Sample: I = 51; C = 52<br>Loss to follow-up: 30<br>Mean age (years): Overall = I=54(12.2), C=55(13.1)

	Gender [Male n (%)]: I = 11 (30.6%); C = 7 (18.9%)<br>Relationship of caregiver to care recipient: spouse I = 42%; c=51%<br>Ethnicity: Caucasian/White I=86%, C=84%<br>SES status: Had at least a high school education I=86% and C=97%
**Intervention**	Description of Intervention: The intervention was constructed with four interrelated components for carers: (1) linked Web sites about stroke and caring; (2) customised educational information or tips specific to carers' needs; (3) an email forum to ask a nurse specialist and a rehabilitation team (therapists, pharmacist, dietitian, social worker and physician) any questions in private and (4) a non-structured email discussion amongst all participants facilitated by the nurse<br>Description of Control: Non-web supported usual care<br>Duration of Intervention: 1 year.<br>Length of follow-up: immediate post (1 year).
**Risk of Bias**<br>**Overall: (U)**	Selection: (U) blocked randomisation scheme with no information on how it was generated.<br>Allocation Concealment: (U) Insufficient information for assessment<br>Blinding Participants: (U) Insufficient information for assessment<br>Blinding Assessors: (U) Insufficient information for assessment<br>Incomplete Data: (H) 30% attrition<br>Selective Reporting: (L) No concerns<br>Other: (L) No concerns

**Study/Location**	**Smith, 2012, US [45]**
**Purpose**	To develop and test the efficacy of a Web-based intervention for alleviating depression in male stroke survivors (SSs) and their spousal caregivers (CGs) that blends both peer and professional support.
**Methods**	Design: RCT<br>Setting /Recruitment Methods: Dyads were recruited nationally through notices on Web sites and listserv announcements of key organizations (e.g., National Stroke Association; Family Caregiver Alliance).<br>Inclusion criteria: the female CG provided care at home to a husband after stroke; either the SS or CG scored five or more on the PHQ-9 (at least

	mild depression); neither SS nor CG were medically unstable or terminally ill; and both were cognitively able to participate.
**Participants**	Recruited Sample: 38<br>Baseline Sample: I = 19; C = 19<br>Loss to follow-up: 3<br>Mean age (years): I = 55.3 (6.9); C = 54.9 (12.9)<br>Gender [Male n (%)]: 100% Female<br>Relationship of caregiver to care recipient: wives of care recipient<br>Ethnicity: primarily Caucasian<br>SES status: <$20,000: Intervention = 4 (28.6); Control = 3 (18.8)<br>$21,000–$35,999: Intervention = 6 (42.8); Control = 8 (50.0)<br>$36,000–$50,999: Intervention = 0 (0); Control = 3 (18.8)<br>$60,000: Intervention = 4 (28.6); Control = 2 (12.5)
**Intervention**	Description of Intervention: The intervention consisted of five components designed to provide CGs with knowledge, resources, and skills to help them both reduce their personal distress and to provide optimal emotional care to the SS: Professional Guide, Educational Videos, Online Chat Sessions, E-mail and Message Board, and Resource Room.<br>Description of Control: Those CGs assigned to this condition had access to the Resource Room only. At the RCT outset, they were asked to watch an online video in which the same Professional Guide explained the features of the Resource Room and encouraged CGs to use it as a caregiving resource. There was no further exposure to the Professional Guide beyond that video. A weekly caregiving tip was also presented online, but none overlapped with content covered in the intervention condition. A toll free phone number was provided in case CGs encountered technological problems while accessing the Resource Room, or if a medical emergency occurred. Halfway through the RCT, an assistant phoned CGs to see if they encountered technical difficulties in accessing the Resource Room. Participants in both RCT conditions received identical computer resources for accessing Web-based information and support. The critical difference was that the control condition had no exposure to the key intervention components.

	Duration of Intervention: 11 weeks<br>Length of follow-up: Immediate post and 1 month follow-up
**Risk of Bias Overall: (L)**	Selection: (L) Computer generated<br>Allocation Concealment: (U) Insufficient information for assessment<br>Blinding Participants: (U) Insufficient information for assessment<br>Blinding Assessors: (L) Blinded assessors<br>Incomplete Data: (L) No concerns<br>Selective Reporting: (L) No concerns<br>Other: (L) no concerns

**Study/Location**	**Torkamani, 2014, UK [41]**
**Purpose**	The current study is a multi-center randomized controlled evaluation of a technology platform specifically designed for PwD living at home and their carers.
**Methods**	Design: RCT<br>Setting/Recruitment Methods: Hospital outpatients identified as having dementia were screened for functional dependency and cognitive impairment using the Barthel Index.<br>Inclusion criteria: Patients living at home with a full time carer, a BI score of at least 35 (indicating some degree of independence), and a MMSE score of at least 9 and no more than 21 (indicating moderate to mild cognitive impairment) were recruited. Patients either had dementia as their primary condition or dementia as part of Parkinson’s disease.
**Participants**	Recruited Sample: 60<br>Baseline Sample: I = 30; C = 30<br>Loss to follow-up: NR<br>Mean age (years): Overall, 60.69 (13.09)<br>Gender [Male n (%)]: NR<br>Relationship of caregiver to care recipient: NR<br>Ethnicity: NR<br>SES status: NR

**Intervention**	Description of Intervention: ALADDIN is a computerized platform designed to offer avenues of support and information to the carer. It also manages and communicates information related to the PwD and their carers from their home to the clinicians, facilitating distant monitoring. ALADDIN has four key features: ‘ALADDIN TV’, ‘SOCIAL NETWORKING’, ‘MY TASKS’, and ‘CONTACT US’.<br>Description of Control: The participants in the control group were only assessed at the three time points, without any further contact or intervention.<br>Duration of Intervention: 6 months<br>Length of follow-up: immediate post (6 months)
**Risk of Bias**<br>**Overall: (U)**	Selection: (U) Insufficient information for assessment<br>Allocation Concealment: (U) Insufficient information for assessment<br>Blinding Participants: (U) Insufficient information for assessment<br>Blinding Assessors: (U) Insufficient information for assessment<br>Incomplete Data: (L) No concerns<br>Selective Reporting: (L) No concerns<br>Other: (L) No concerns
