# Supplementary material for: Impact of Internet-Based Interventions on Caregiver Mental Health: Systematic Review and Meta-Analysis
Source: J Med Internet Res. 2018 Jul 3;20(7):e10668. doi: 10.2196/10668 (PMC6053616; doi:10.2196/10668)
Supplement: Multimedia Appendix 3 [file jmir_v20i7e10668_app3.pdf]

Multimedia Appendix 3. Mental health outcomes and measurement tools

Outcome	Measurement Tool
Depression	Beck Depression Inventory (BDI-II) [39]<br>Centre for Epidemiological Studies Depression Scale (CES-D) [37] [38] [44] [45] [46] [48] [49]
Stress or Distress	Perceived Stress Scale (PSS) [39] [44]<br>Neuropsychiatric Inventory (NPI) [41]<br>Customized two question approach [48]<br>Interpersonal Reactivity Index (IRI) [40]
Anxiety	Hospital Anxiety and Depression Scale (HADS) [38]<br>State-Trait Anxiety Inventory (STAI) [48]
Coping	Brief Cope Scale (BCS) [43]<br>Revised Ways of Coping (R-WoC) [48]
Mental Health	Short Version Profile of Mood States (SV-POMS) [42]
Quality of Life	Quality of Life in Alzheimer's Disease Scale (QoLAD)[a] [50]<br>Non-specific tool (2 questions) [40]<br>Quality of Life Scale (QoLS) [41]<br>Perceived Quality of Life (PQoL) [44]
Overall Health	Nottingham Health Profile (NHP) [39]<br>EuroQoL (EQ5D) [41]

[a] Plus, the Minimum Dataset of the Dutch National Programme for Elderly Care (MDS-NPO).
