# Supplementary material for: Impact of Internet-Based Interventions on Caregiver Mental Health: Systematic Review and Meta-Analysis
Source: J Med Internet Res. 2018 Jul 3;20(7):e10668. doi: 10.2196/10668 (PMC6053616; doi:10.2196/10668)

Multimedia Appendix4: Meta-analysis and forest plots

Mental Health and General/Overall Health outcomes

Change in Depression

Study or Subgroup	Experimental Mean	Experimental SD	Experimental Total	Control Mean	Control SD	Control Total	Weight	Std. Mean Difference IV, Random, 95% CI
**1.1.1 Internet: Information / Education**								
Beauchamp, 2005	-1.4	7.574	150	1	7.819	149	19.3%	-0.31 [-0.54, -0.08]
Kajiyama, 2013	-2.52	5.532	46	-0.77	6.215	57	14.4%	-0.29 [-0.68, 0.10]
Núñez-Naveira, 2016	-2.37	5.98	30	-0.65	6.254	31	11.5%	-0.28 [-0.78, 0.23]
**Subtotal (95% CI)**			**226**			**237**	**45.2%**	**-0.30 [-0.49, -0.12]**
Heterogeneity: Tau² = 0.00; Chi² = 0.02, df = 2 (P = 0.99); I² = 0%								
Test for overall effect: Z = 3.23 (P = 0.001)								
**1.1.2 Internet: Information/ Education + Psychosocial support - Peer**								
Cristancho-Lacroix, 2015	0.3	4.6	25	-0.1	2.7	24	10.3%	0.10 [-0.46, 0.66]
**Subtotal (95% CI)**			**25**			**24**	**10.3%**	**0.10 [-0.46, 0.66]**
Heterogeneity: Not applicable								
Test for overall effect: Z = 0.36 (P = 0.72)								
**1.1.3 Internet: Information/ Education + Psychosocial support - Professional**								
Blom, 2015	-2.35	8.21	90	0.34	7.51	90	17.3%	-0.34 [-0.63, -0.05]
**Subtotal (95% CI)**			**90**			**90**	**17.3%**	**-0.34 [-0.63, -0.05]**
Heterogeneity: Not applicable								
Test for overall effect: Z = 2.27 (P = 0.02)								
**1.1.5 Internet: Information/ Education + Psychosocial support - Peers + Psychosocial support - Professional**								
Pagan-Ortiz, 2014	0.02	0.403	15	-0.1	0.35	17	7.8%	0.31 [-0.39, 1.01]
Pierce, 2009	0.3	6.966	36	-2.3	6.222	37	12.5%	0.39 [-0.07, 0.85]
Smith, 2012	-7.8	8.993	15	2	7.856	17	7.0%	-1.14 [-1.89, -0.38]
**Subtotal (95% CI)**			**66**			**71**	**27.3%**	**-0.11 [-1.01, 0.78]**
Heterogeneity: Tau² = 0.52; Chi² = 12.08, df = 2 (P = 0.002); I² = 83%								
Test for overall effect: Z = 0.25 (P = 0.80)								
**Total (95% CI)**			**407**			**422**	**100.0%**	**-0.19 [-0.43, 0.05]**
Heterogeneity: Tau² = 0.06; Chi² = 17.23, df = 7 (P = 0.02); I² = 59%								
Test for overall effect: Z = 1.55 (P = 0.12)								
Test for subgroup differences: Chi² = 2.15, df = 3 (P = 0.54), I² = 0%								

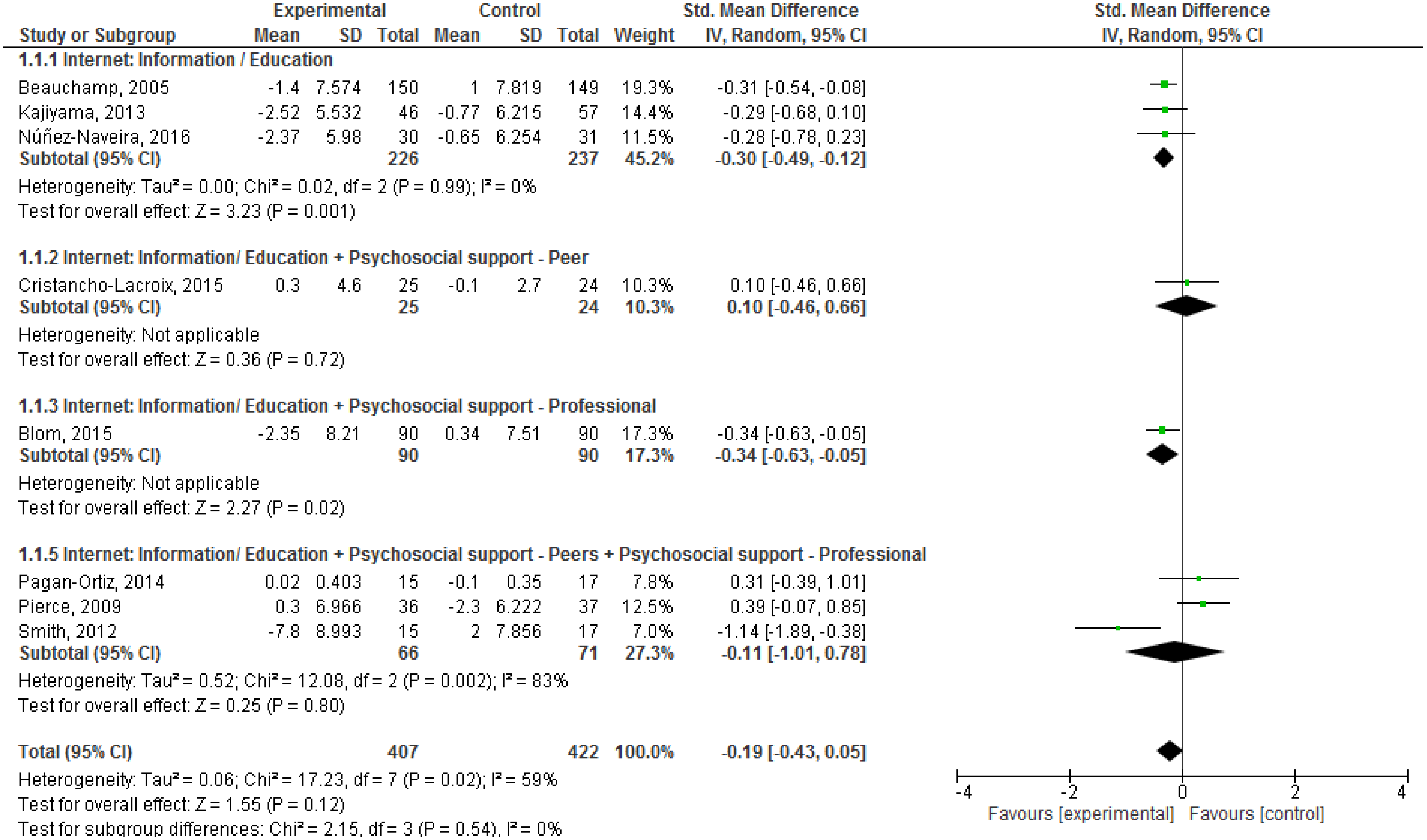

Change in Stress / Distress

Study or Subgroup	Experimental Mean	Experimental SD	Experimental Total	Control Mean	Control SD	Control Total	Weight	Std. Mean Difference IV, Random, 95% CI
**1.2.1 Internet: Information / Education**								
Beauchamp, 2005	-3.4	5.111	150	-0.7	4.771	149	28.5%	-0.54 [-0.78, -0.31]
Kajiyama, 2013	-2.63	3.633	46	0.19	4.964	57	20.1%	-0.63 [-1.03, -0.23]
**Subtotal (95% CI)**			**196**			**206**	**48.6%**	**-0.57 [-0.77, -0.37]**
Heterogeneity: $Tau^2 = 0.00$; $Chi^2 = 0.14$, df = 1 (P = 0.71); $I^2 = 0\%$								
Test for overall effect: Z = 5.56 (P < 0.00001)								
**1.2.2 Internet: Information/ Education + Psychosocial support - Peer**								
Cristancho-Lacroix, 2015	-0.5	8	25	-0.7	4.5	24	14.0%	0.03 [-0.53, 0.59]
Hattink, 2015	-4.59	4.157	27	-0.66	4.064	32	14.6%	-0.94 [-1.49, -0.40]
**Subtotal (95% CI)**			**52**			**56**	**28.6%**	**-0.46 [-1.41, 0.50]**
Heterogeneity: $Tau^2 = 0.40$; $Chi^2 = 6.01$, df = 1 (P = 0.01); $I^2 = 83\%$								
Test for overall effect: Z = 0.94 (P = 0.35)								
**1.2.3 Internet: Information/ Education + Psychosocial support - Peers + Psychosocial support - Professional**								
Marziali, 2006	-1.326	5.44	23	2.519	5.44	15	11.1%	-0.69 [-1.36, -0.02]
Torkamani, 2014	-0.38	4.042	17	-0.65	3.389	20	11.6%	0.07 [-0.58, 0.72]
**Subtotal (95% CI)**			**40**			**35**	**22.7%**	**-0.30 [-1.05, 0.44]**
Heterogeneity: $Tau^2 = 0.18$; $Chi^2 = 2.58$, df = 1 (P = 0.11); $I^2 = 61\%$								
Test for overall effect: Z = 0.80 (P = 0.42)								
**Total (95% CI)**			**288**			**297**	**100.0%**	**-0.48 [-0.75, -0.22]**
Heterogeneity: $Tau^2 = 0.05$; $Chi^2 = 9.88$, df = 5 (P = 0.08); $I^2 = 49\%$								
Test for overall effect: Z = 3.54 (P = 0.0004)								
Test for subgroup differences: $Chi^2 = 0.47$, df = 2 (P = 0.79), $I^2 = 0\%$								

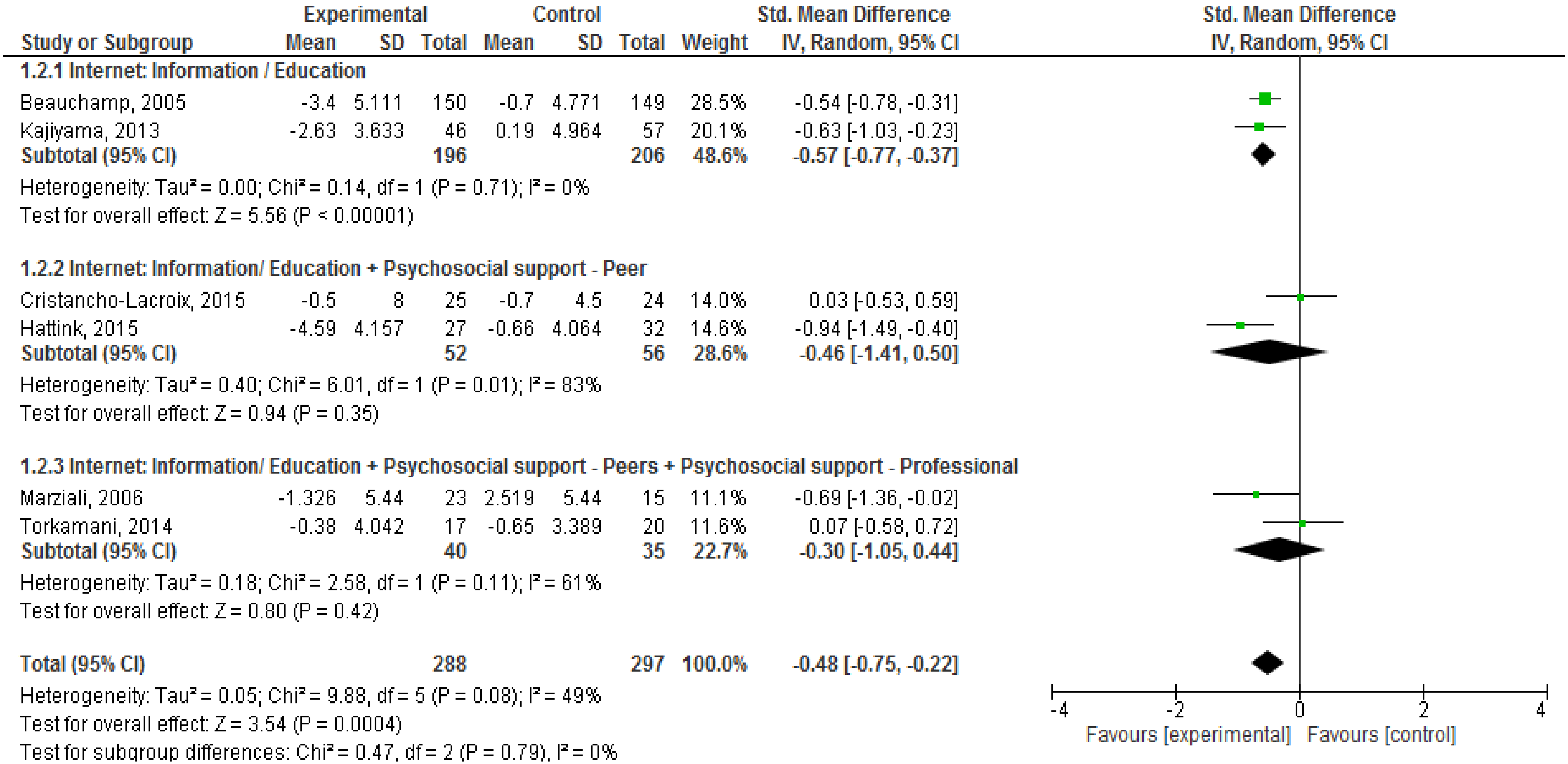

Change in Anxiety

	Experimental			Control				Std. Mean Difference
Study or Subgroup	Mean	SD	Total	Mean	SD	Total	Weight	IV, Random, 95% CI
**1.3.1 Internet: Information / Education**								
Beauchamp, 2005	-0.9	4.035	150	0.9	4.525	149	62.3%	-0.42 [-0.65, -0.19]
**Subtotal (95% CI)**			**150**			**149**	**62.3%**	**-0.42 [-0.65, -0.19]**
Heterogeneity: Not applicable								
Test for overall effect: Z = 3.58 (P = 0.0003)								
**1.3.2 Internet: Information/ Education + Psychosocial support - Professional**								
Blom, 2015	-1.69	3.26	90	-0.47	3.41	90	37.7%	-0.36 [-0.66, -0.07]
**Subtotal (95% CI)**			**90**			**90**	**37.7%**	**-0.36 [-0.66, -0.07]**
Heterogeneity: Not applicable								
Test for overall effect: Z = 2.42 (P = 0.02)								
**Total (95% CI)**			**240**			**239**	**100.0%**	**-0.40 [-0.58, -0.22]**
Heterogeneity: Tau² = 0.00; Chi² = 0.08, df = 1 (P = 0.77); I² = 0%								
Test for overall effect: Z = 4.31 (P < 0.0001)								
Test for subgroup differences: Chi² = 0.08, df = 1 (P = 0.77), I² = 0%								

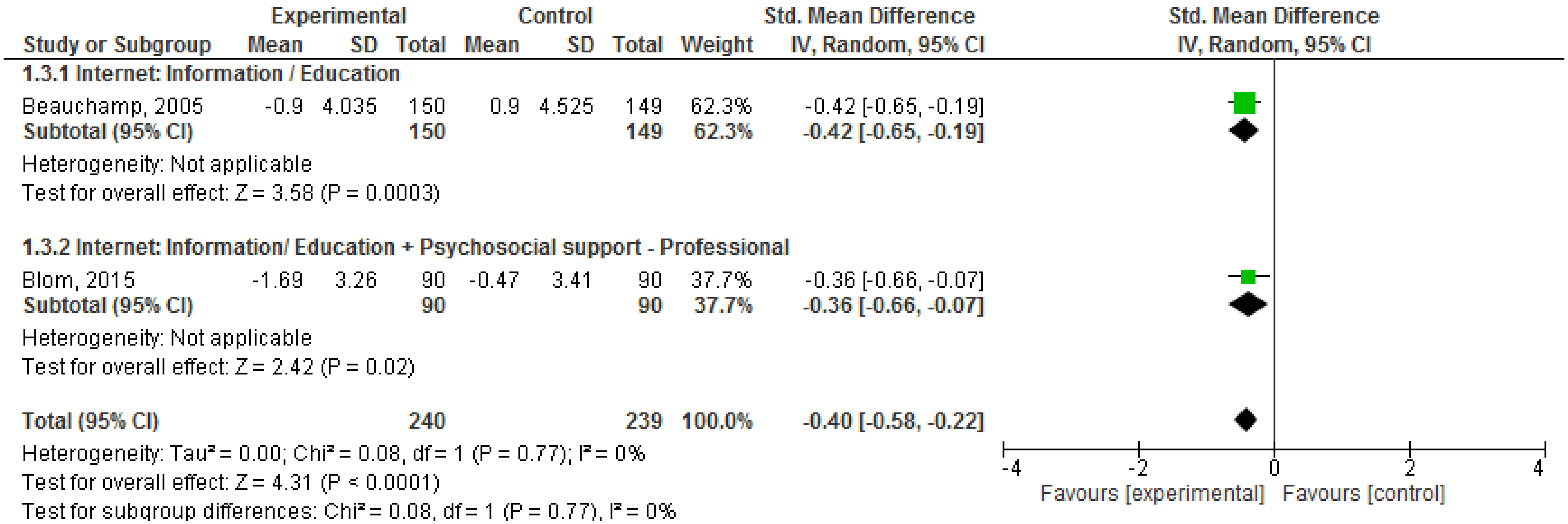

Change in Coping

	Experimental			Control				Std. Mean Difference
Study or Subgroup	Mean	SD	Total	Mean	SD	Total	Weight	IV, Random, 95% CI
**1.4.1 Internet: Information / Education**								
Beauchamp, 2005	1	8.039	150	1	7.881	149	74.3%	0.00 [-0.23, 0.23]
**Subtotal (95% CI)**			**150**			**149**	**74.3%**	**0.00 [-0.23, 0.23]**
Heterogeneity: Not applicable								
Test for overall effect: Z = 0.00 (P = 1.00)								
**1.4.2 Internet: Information/ Education + Psychosocial support - Peers + Psychosocial support - Professional**								
Namkoong, 2012	-0.42	0.655	49	-0.4	0.713	55	25.7%	-0.03 [-0.41, 0.36]
**Subtotal (95% CI)**			**49**			**55**	**25.7%**	**-0.03 [-0.41, 0.36]**
Heterogeneity: Not applicable								
Test for overall effect: Z = 0.15 (P = 0.88)								
**Total (95% CI)**			**199**			**204**	**100.0%**	**-0.01 [-0.20, 0.19]**
Heterogeneity: Tau² = 0.00; Chi² = 0.02, df = 1 (P = 0.90); I² = 0%								
Test for overall effect: Z = 0.07 (P = 0.94)								
Test for subgroup differences: Chi² = 0.02, df = 1 (P = 0.90), I² = 0%								

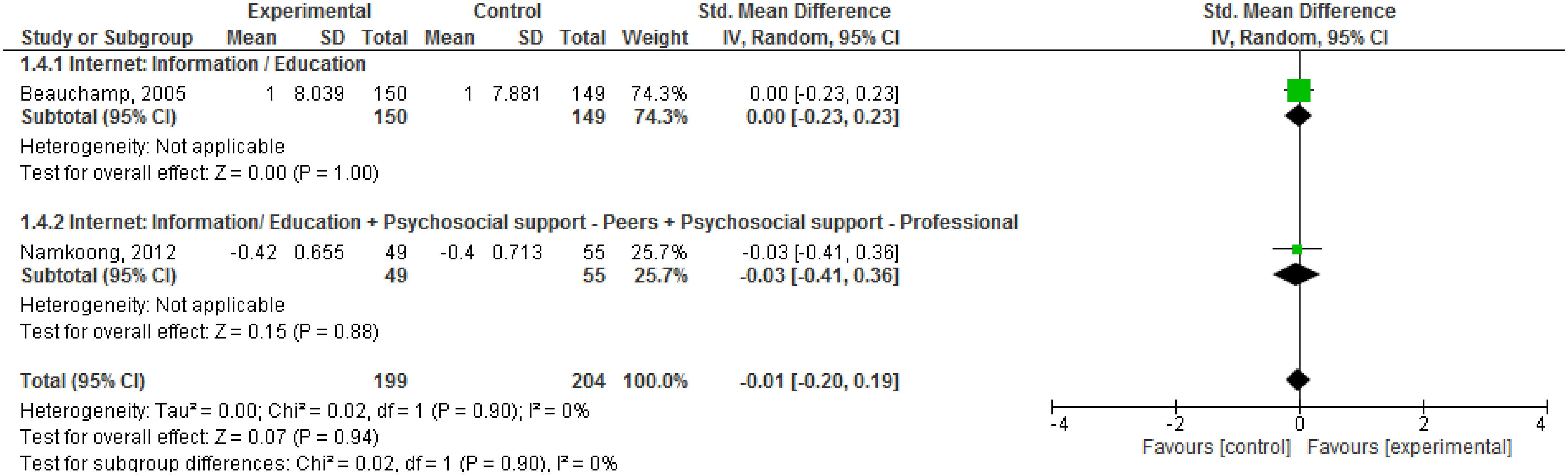

Change in Overall Mental health

	Experimental			Control				Std. Mean Difference
Study or Subgroup	Mean	SD	Total	Mean	SD	Total	Weight	IV, Random, 95% CI
**1.5.1 Internet: Information/ Education + Psychosocial support - Peers + Psychosocial support - Professional**								
DuBenske, 2014	-0.23	0.51	45	-0.06	0.637	52	100.0%	-0.29 [-0.69, 0.11]
**Subtotal (95% CI)**			**45**			**52**	**100.0%**	**-0.29 [-0.69, 0.11]**
Heterogeneity: Not applicable								
Test for overall effect: Z = 1.42 (P = 0.16)								
**Total (95% CI)**			**45**			**52**	**100.0%**	**-0.29 [-0.69, 0.11]**
Heterogeneity: Not applicable								
Test for overall effect: Z = 1.42 (P = 0.16)								
Test for subgroup differences: Not applicable								

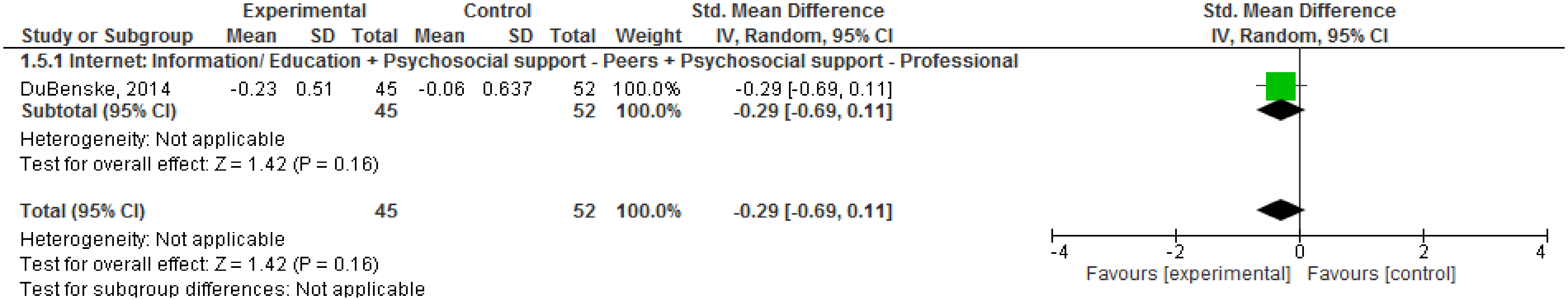

Change in Quality of life

Study or Subgroup	Experimental Mean	Experimental SD	Experimental Total	Control Mean	Control SD	Control Total	Weight	Std. Mean Difference IV, Random, 95% CI
**2.1.1 Internet: Information / Education**								
Kajiyama, 2013	0.49	1.228	46	0.06	1.323	57	30.6%	0.33 [-0.06, 0.72]
**Subtotal (95% CI)**			**46**			**57**	**30.6%**	**0.33 [-0.06, 0.72]**
Heterogeneity: Not applicable								
Test for overall effect: Z = 1.67 (P = 0.10)								
**2.1.2 Internet: Information/ Education + Psychosocial support - Peer**								
Hattink, 2015	-0.19	1.198	21	0.25	1.188	25	24.9%	-0.36 [-0.95, 0.22]
**Subtotal (95% CI)**			**21**			**25**	**24.9%**	**-0.36 [-0.95, 0.22]**
Heterogeneity: Not applicable								
Test for overall effect: Z = 1.21 (P = 0.22)								
**2.1.3 Internet: Information/ Education + Psychosocial support - Peers + Psychosocial support - Professional**								
Torkamani, 2014	15.45	9.132	18	10.5	8.372	20	23.1%	0.55 [-0.10, 1.20]
**Subtotal (95% CI)**			**18**			**20**	**23.1%**	**0.55 [-0.10, 1.20]**
Heterogeneity: Not applicable								
Test for overall effect: Z = 1.67 (P = 0.09)								
**2.1.4 Internet + telephone: Monitoring + Psychosocial support – Peers + Psychosocial support - Professional**								
Hattink, 2016	-1.64	5.376	17	1.13	3.213	15	21.4%	-0.60 [-1.31, 0.11]
**Subtotal (95% CI)**			**17**			**15**	**21.4%**	**-0.60 [-1.31, 0.11]**
Heterogeneity: Not applicable								
Test for overall effect: Z = 1.65 (P = 0.10)								
**Total (95% CI)**			**102**			**117**	**100.0%**	**0.01 [-0.49, 0.51]**

Heterogeneity: $Tau^2 = 0.18$; $Chi^2 = 9.35$, df = 3 (P = 0.02); $I^2 = 68\%$
Test for overall effect: Z = 0.04 (P = 0.97)
Test for subgroup differences: $Chi^2 = 9.35$, df = 3 (P = 0.02), $I^2 = 67.9\%$

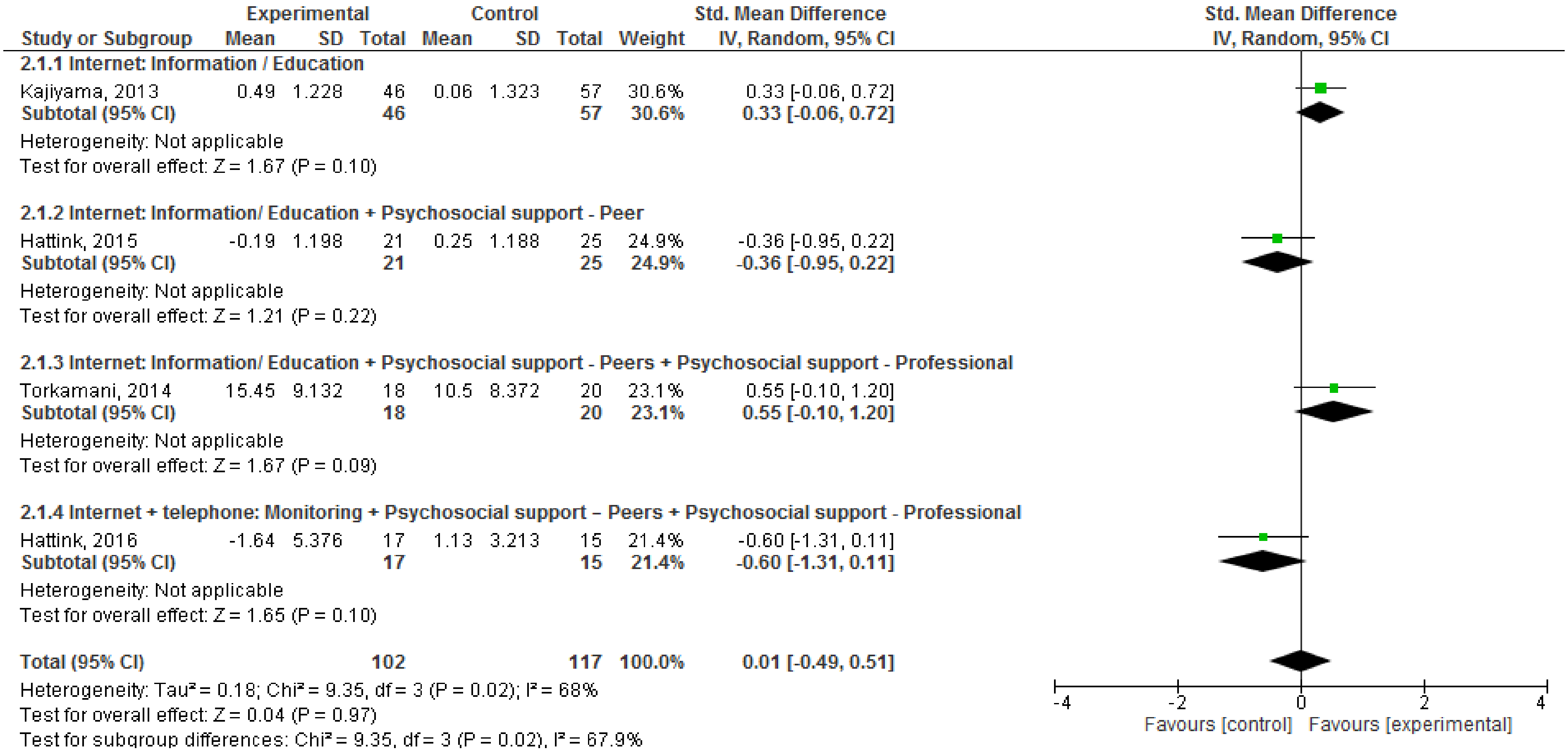

Change in Overall Health

Study or Subgroup	Experimental Mean	Experimental SD	Experimental Total	Control Mean	Control SD	Control Total	Weight	Std. Mean Difference IV, Random, 95% CI
**2.2.1 Internet: Information/ Education + Psychosocial support - Peer**								
Cristancho-Lacroix, 2015	-2.6	30.6	25	11.9	34.2	24	53.1%	-0.44 [-1.01, 0.13]
**Subtotal (95% CI)**			**25**			**24**	**53.1%**	**-0.44 [-1.01, 0.13]**
Heterogeneity: Not applicable								
Test for overall effect: Z = 1.52 (P = 0.13)								
**2.2.2 Internet: Information/ Education + Psychosocial support - Peers + Psychosocial support - Professional**								
Torkamani, 2014	0.15	0.219	9	-0.1	0.163	10	46.9%	1.25 [0.24, 2.25]
**Subtotal (95% CI)**			**9**			**10**	**46.9%**	**1.25 [0.24, 2.25]**
Heterogeneity: Not applicable								
Test for overall effect: Z = 2.43 (P = 0.01)								
**Total (95% CI)**			**34**			**34**	**100.0%**	**0.35 [-1.30, 2.00]**
Heterogeneity: Tau² = 1.25; Chi² = 8.22, df = 1 (P = 0.004); I² = 88%								
Test for overall effect: Z = 0.42 (P = 0.68)								
Test for subgroup differences: Chi² = 8.22, df = 1 (P = 0.004), I² = 87.8%								

Std. Mean Difference
IV, Random, 95% CI

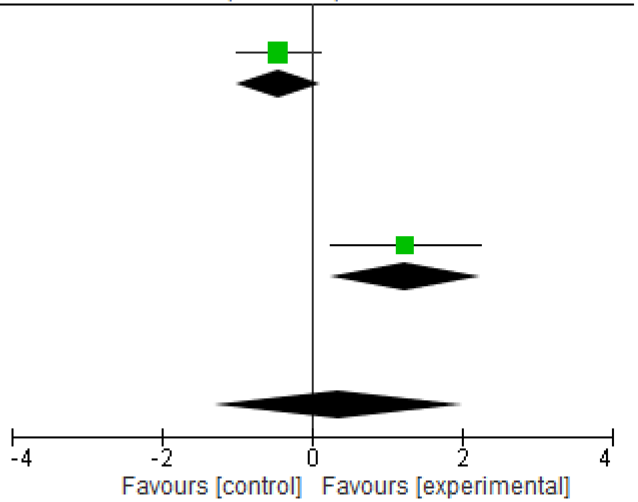

Supplement: Multimedia Appendix 4 [file jmir_v20i7e10668_app4.pdf]
