# Supplementary material for: Impact of Internet-Based Interventions on Caregiver Mental Health: Systematic Review and Meta-Analysis
Source: J Med Internet Res. 2018 Jul 3;20(7):e10668. doi: 10.2196/10668 (PMC6053616; doi:10.2196/10668)
Supplement: Multimedia Appendix 5 [file jmir_v20i7e10668_app5.pdf]

Multimedia Appendix 5: Detailed GRADE Evidence Tables

Table 1: Grade table for any internet-based interventions

Patient or population: Caregivers
Intervention: Internet-based interventions
Comparison: Control

Outcomes	Anticipated absolute effects[*] (95% CI)		Relative effect (95% CI)	№ of participants (studies)	Quality of the evidence (GRADE)	Comments
	Risk with Control	Risk with eTechnology based interventions				
Change in Depression	-	SMD **0.19 SD lower** (0.43 lower to 0.05 higher)	-	829 (8 RCTs) [a]	⊕◯◯◯ VERY LOW [b,c,d,e]	Assessed using 20-item Center for Epidemiologic Studies Depression Scale (range: 0-60) in 7 studies and 21-item Beck Depression Inventory (range: 0-63) in one study.
Change in Stress / Distress	-	SMD **0.48 SD lower** (0.75 lower to 0.22 lower)	-	585 (6 RCTs) [f]	⊕⊕◯◯ LOW [b,d]	Assessed using Perceived Stress Scale, Interpersonal Reactivity Index, Neuropsychiatric Inventory, Perceived Stress Scale and 2 items stress scale across studies.
Change in Anxiety	-	SMD **0.4 SD lower** (0.58 lower to 0.22 lower)	-	479 (2 RCTs) [g]	⊕⊕◯◯ LOW [b,d]	Assessed using 10-item State–Trait Anxiety Inventory (range:0-40) in one study and 7-item Hospital Anxiety and Depression Scale (0-21) in the other study.
Change in Coping	-	SMD **0.01 SD lower** (0.2 lower to 0.19 higher)	-	403 (2 RCTs) [h]	⊕◯◯◯ VERY LOW [b,d,e]	Assessed using 15-item Revised Ways of Coping scale (range: 15-60) in one study and 5-point Likert type Brief Cope scale in the other study.

Table 1: Grade table for any internet-based interventions

Patient or population: Caregivers
Intervention: Internet-based interventions
Comparison: Control

Outcomes	**Anticipated absolute effects**[*] (95% CI)		Relative effect (95% CI)	№ of participants (studies)	Quality of the evidence (GRADE)	Comments
	**Risk with Control**	**Risk with eTechnology based interventions**				
Change in Overall Mental health	-	SMD **0.29 SD lower** (0.69 lower to 0.11 higher)	-	97 (1 RCT) [i]	⨁◯◯◯ VERY LOW [b,j]	Assessed using 16-item subset of negative mood items from the Short Version Profile of Mood States (SV-POMS). Likert-type items are rated on scales from 0–4.
Change in Quality of life	-	SMD **0.01 SD higher** (0.49 lower to 0.51 higher)	-	219 (4 RCTs) [k]	⨁◯◯◯ VERY LOW [b,d,j]	Assessed using 19-item Perceived quality of life, 15-item Quality of Life in Alzheimer's Disease Informal caregivers, 2-item Quality of Life scale, and Quality of Life questionnaire across studies.
Change in Overall Health	-	SMD **0.35 SD higher** (1.3 lower to 2 higher)	-	68 (2 RCTs) [l]	⨁◯◯◯ VERY LOW [b,d,j]	Assessed using Nottingham Health Profile (range: 0-100) in one study and EuroQoL, 5 item questionnaire covering five dimensions of QoL in the other study.

Quality assessment							№ of patients		Effect		Quality	Importance
**№ of studies**	**Study design**	**Risk of bias**	**Inconsistency**	**Indirectness**	**Imprecision**	**Other considerations**	**eTechnology based interventions**	**Control**	**Relative (95% CI)**	**Absolute (95% CI)**		
Change in Depression												
8 [a]	randomised trials	serious [b]	serious [c]	serious [d]	serious [e]	none	407	422	-	SMD **0.19 SD lower** (0.43 lower to 0.05 higher)	⊕◯◯◯ VERY LOW	CRITICAL
Change in Stress / Distress												
6 [f]	randomised trials	serious [b]	not serious	serious [d]	not serious	none	288	297	-	SMD **0.48 SD lower** (0.75 lower to 0.22 lower)	⊕⊕◯◯ LOW	CRITICAL
Change in Anxiety												
2 [g]	randomised trials	serious [b]	not serious	serious [d]	not serious	none	240	239	-	SMD **0.4 SD lower** (0.58 lower to 0.22 lower)	⊕⊕◯◯ LOW	CRITICAL
Change in Coping												

Quality assessment							№ of patients		Effect		Quality	Importance
**№ of studies**	**Study design**	**Risk of bias**	**Inconsistency**	**Indirectness**	**Imprecision**	**Other considerations**	**eTechnology based interventions**	**Control**	**Relative (95% CI)**	**Absolute (95% CI)**		
2 [h]	randomised trials	serious [b]	not serious	serious [d]	serious [e]	none	199	204	-	SMD **0.01 SD lower** (0.2 lower to 0.19 higher)	⨁◯◯◯ VERY LOW	CRITICAL
Change in Overall Mental health												
1 [i]	randomised trials	serious [b]	not serious	not serious	very serious [j]	none	45	52	-	SMD **0.29 SD lower** (0.69 lower to 0.11 higher)	⨁◯◯◯ VERY LOW	CRITICAL
Change in Quality of life												
4 [k]	randomised trials	serious [b]	not serious	serious [d]	very serious [j]	none	102	117	-	SMD **0.01 SD higher** (0.49 lower to 0.51 higher)	⨁◯◯◯ VERY LOW	CRITICAL
Change in Overall Health												

Quality assessment							№ of patients		Effect		Quality	Importance
№ of studies	Study design	Risk of bias	Inconsistency	Indirectness	Imprecision	Other considerations	eTechnology based interventions	Control	Relative (95% CI)	Absolute (95% CI)		
2 [l]	randomised trials	serious [b]	not serious	serious [d]	very serious [j]	none	34	34	-	SMD **0.35 SD higher** (1.3 lower to 2 higher)	⨁◯◯◯ VERY LOW	CRITICAL

CI: Confidence interval; **SMD:** Standardised mean difference

Explanations

a. 1) Beauchamp, 2005; 2) Kajiyama, 2013; 3) Núñez-Naveira, 2016; 4) Cristancho-Lacroix, 2015; 5) Blom, 2015; 6) Pagan-Ortiz, 2014; 7) Pierce, 2009; 8) Smith, 2012.
b. Serious concerns regarding risk of bias.
c. The confidence intervals do not overlap across studies and statistical heterogeneity is moderate (I-squared = 59%; p = 0.02).
d. Serious concerns regarding clinical/methodological heterogeneity across studies due to differences in type and focus of e-technology interventions, length of intervention and informal caregiver population.
e. The effect estimate is imprecise.
f. 1) Beauchamp, 2005; 2) Kajiyama, 2013; 3) Cristancho-Lacroix, 2015; 4) Hattink, 2015; 5) Marziali, 2006; 6) Torkamani, 2014.
g. 1) Beauchamp, 2005; 2) Blom, 2015.
h. 1) Beauchamp, 2005; 2) Namkoong, 2012.
i. DuBenske, 2014
j. The sample size is <300 and effect estimate is imprecise.
k. 1) Kajiyama, 2013; 2) Hattink, 2015; 3) Torkamani, 2014; 4) Hattink, 2016
l. 1) Cristancho-Lacroix, 2015; 2) Torkamani, 2014.

Table 2: Grade table for any internet-based information or education only intervention

Patient or population: Caregivers
Intervention: Technology (Internet: Information / Education only)
Comparison: Control

Outcomes	Anticipated absolute effects* (95% CI)		Relative effect (95% CI)	№ of participants (studies)	Quality of the evidence (GRADE)	Comments
	Risk with Control	Risk with Technology (Internet: Information / Education only)				
Change in Depression	-	SMD **0.31 SD lower** (0.50 lower to 0.11 lower)	-	402 (2 RCTs) [a]	⊕⊕⊕◯ MODERATE [b]	Assessed using Center for Epidemiologic Studies Depression Scale: CES-D consisting of 20 items. The total score range is 0 to 60.
Change in Stress / Distress	-	SMD **0.57 SD lower** (0.77 lower to 0.37 lower)	-	402 (2 RCTs) [c]	⊕⊕⊕◯ MODERATE [b]	Assessed using 10-item Perceived Stress scale (range: 0 to 30) in one study and 2-item Stress scale (range: 0 to 9) in the other study.
Change in Anxiety	-	SMD **0.42 SD lower** (0.65 lower to 0.19 lower)	-	299 (1 RCT) [d]	⊕⊕⊕◯ MODERATE [b]	Assessed using 10-item subscale of the State–Trait Anxiety Inventory on a 4-point Likert scale (range: 0 to 30), from 3 (very much so) to 0 (not at all).
Change in coping	-	SMD **0 SD** (0.23 lower to 0.23 higher)	-	299 (1 RCT) [d]	⊕⊕◯◯ LOW [e,f]	Assessed using Revised Ways of Coping scale, problem-focused strategies (15 items, range: 15 to 60) on 4-point Likert scale from 1 (never used) to 4 (regularly used).

Table 2: Grade table for any internet-based information or education only intervention

Patient or population: Caregivers
Intervention: Technology (Internet: Information / Education only)
Comparison: Control

Outcomes	Anticipated absolute effects* (95% CI)		Relative effect (95% CI)	№ of participants (studies)	Quality of the evidence (GRADE)	Comments
	Risk with Control	Risk with Technology (Internet: Information / Education only)				
Change in Quality of life	-	SMD **0.33 SD higher** (0.06 lower to 0.72 higher)	-	103 (1 RCT) [g]	⨁◯◯◯ VERY LOW [e,f]	Assessed using Perceived quality of life (PQoL) with 19 items describing level of satisfaction.

***The risk in the intervention group** (and its 95% confidence interval) is based on the assumed risk in the comparison group and the **relative effect** of the intervention (and its 95% CI).

CI: Confidence interval; **SMD:** Standardised mean difference

GRADE Working Group grades of evidence
High quality: We are very confident that the true effect lies close to that of the estimate of the effect
Moderate quality: We are moderately confident in the effect estimate: The true effect is likely to be close to the estimate of the effect, but there is a possibility that it is substantially different
Low quality: Our confidence in the effect estimate is limited: The true effect may be substantially different from the estimate of the effect
Very low quality: We have very little confidence in the effect estimate: The true effect is likely to be substantially different from the estimate of effect

Quality assessment							№ of patients		Effect		Quality	Importance
**№ of studies**	**Study design**	**Risk of bias**	**Inconsistency**	**Indirectness**	**Imprecision**	**Other considerations**	**Technology (Internet: Information / Education only)**	**Control**	**Relative (95% CI)**	**Absolute (95% CI)**		
Change in Depression												
2 [a]	randomised trials	serious [b]	not serious	not serious	not serious	none	196	206	-	SMD **0.31 SD lower** (0.50 lower to 0.11 lower)	⨁⨁⨁◯ MODERATE	CRITICAL
Change in Stress / Distress												
2 [c]	randomised trials	serious [b]	not serious	not serious	not serious	none	196	206	-	SMD **0.57 SD lower** (0.77 lower to 0.37 lower)	⨁⨁⨁◯ MODERATE	CRITICAL
Change in Anxiety												
1 [d]	randomised trials	serious [b]	not serious	not serious	not serious	none	150	149	-	SMD **0.42 SD lower** (0.65 lower to 0.19 lower)	⨁⨁⨁◯ MODERATE	CRITICAL

Quality assessment							№ of patients		Effect		Quality	Importance
**№ of studies**	**Study design**	**Risk of bias**	**Inconsistency**	**Indirectness**	**Imprecision**	**Other considerations**	**Technology (Internet: Information / Education only)**	**Control**	**Relative (95% CI)**	**Absolute (95% CI)**		
Change in coping												
1 [d]	randomised trials	serious [e]	not serious	not serious	serious [f]	none	150	149	-	SMD **0 SD** (0.23 lower to 0.23 higher)	⨁⨁◯◯ LOW	CRITICAL
Change in Quality of life												
1 [g]	randomised trials	serious [e]	not serious	not serious	very serious [f]	none	46	57	-	SMD **0.33 SD higher** (0.06 lower to 0.72 higher)	⨁◯◯◯ VERY LOW	CRITICAL

CI: Confidence interval; **SMD:** Standardised mean difference

Explanations

a. 1) Kajiyama, 2013; 2) Beauchamp, 2005
b. Serious concerns for risk of bias.
c. 1) Kajiyama, 2013; 2) Beauchamp, 2005
d. Beauchamp, 2005
e. Serious concerns regarding risk of bias.
f. The effect estimate is imprecise.
g. Kajiyama, 2013

Table 3: Grade table for any internet-based information or education intervention + peer psychosocial support

Patient or population: Caregivers
Intervention: Technology (Internet: Information/Education + Peer psychosocial support)
Comparison: Control

Outcomes	Anticipated absolute effects* (95% CI)		Relative effect (95% CI)	№ of participants (studies)	Quality of the evidence (GRADE)	Comments
	Risk with Control	Risk with Technology (Internet: Information/Education + Peer psychosocial support)				
Change in Depression	-	SMD **0.11 SD lower** (0.48 lower to 0.27 higher)	-	110 (2 RCT) [a]	⊕◯◯◯ VERY LOW [b,c]	Assessed using 21-item Beck Depression Inventory (BDI-II; range 0 to 63) in 1 study and 20-item Center for Epidemiologic Studies Depression Scale (CES-D; range 0 to 60) in the other study.
Change in Stress / Distress	-	SMD **0.46 SD lower** (1.41 lower to 0.5 higher)	-	108 (2 RCTs) [d]	⊕◯◯◯ VERY LOW [b,c]	Assessed using 14-item Perceived Stress Scale (PSS-14, range: 0-56) in one study & 28-item Interpersonal Reactivity Index (IRI, 5-point scale, range: 0 to 112) in the other study.
Change in Quality of life	-	SMD **0.36 SD lower** (0.95 lower to 0.22 higher)	-	46 (1 RCT) [e]	⊕◯◯◯ VERY LOW [b,c]	Assessed using 2-item Quality of life scale on a scale from 1 to 10.
Change in Overall health	-	SMD **0.44 SD lower** (1.01 lower to 0.13 higher)	-	49 (1 RCT) [f]	⊕◯◯◯ VERY LOW [b,c]	Assessed using Nottingham Health Profile (NHP) with social isolation, emotional reactions, and sleep quality sub-scores and rated each from 0 to 100.

Table 3: Grade table for any internet-based information or education intervention + peer psychosocial support

Patient or population: Caregivers
Intervention: Technology (Internet: Information/Education + Peer psychosocial support)
Comparison: Control

Outcomes	**Anticipated absolute effects*** (95% CI)		Relative effect (95% CI)	№ of participants (studies)	Quality of the evidence (GRADE)	Comments
	**Risk with Control**	**Risk with Technology (Internet: Information/Education + Peer psychosocial support)**				

*__The risk in the intervention group__ (and its 95% confidence interval) is based on the assumed risk in the comparison group and the **relative effect** of the intervention (and its 95% CI).

CI: Confidence interval; **SMD:** Standardised mean difference

GRADE Working Group grades of evidence
High quality: We are very confident that the true effect lies close to that of the estimate of the effect
Moderate quality: We are moderately confident in the effect estimate: The true effect is likely to be close to the estimate of the effect, but there is a possibility that it is substantially different
Low quality: Our confidence in the effect estimate is limited: The true effect may be substantially different from the estimate of the effect
Very low quality: We have very little confidence in the effect estimate: The true effect is likely to be substantially different from the estimate of effect

**Quality assessment**							**№ of patients**		**Effect**		**Quality**	**Importance**
**№ of studies**	**Study design**	**Risk of bias**	**Inconsistency**	**Indirectness**	**Imprecision**	**Other considerations**	**Technology (Internet: Information/Education + Peer psychosocial support)**	**Control**	**Relative (95% CI)**	**Absolute (95% CI)**		
Change in Depression												

Quality assessment							№ of patients		Effect		Quality	Importance
№ of studies	Study design	Risk of bias	Inconsistency	Indirectness	Imprecision	Other considerations	Technology (Internet: Information/Education + Peer psychosocial support)	Control	Relative (95% CI)	Absolute (95% CI)		
2 [a]	randomised trials	serious [b]	not serious	not serious	very serious [c]	none	55	55	-	SMD **0.11 SD lower** (0.48 lower to 0.27 higher)	⨁◯◯◯ VERY LOW	CRITICAL
Change in Stress / Distress												
2 [d]	randomised trials	serious [b]	not serious	not serious	very serious [c]	none	52	56	-	SMD **0.46 SD lower** (1.41 lower to 0.5 higher)	⨁◯◯◯ VERY LOW	CRITICAL
Change in Quality of life												
1 [e]	randomised trials	serious [b]	not serious	not serious	very serious [c]	none	21	25	-	SMD **0.36 SD lower** (0.95 lower to 0.22 higher)	⨁◯◯◯ VERY LOW	CRITICAL
Change in Overall health												

Quality assessment							№ of patients		Effect		Quality	Importance
№ of studies	Study design	Risk of bias	Inconsistency	Indirectness	Imprecision	Other considerations	Technology (Internet: Information/Education + Peer psychosocial support)	Control	Relative (95% CI)	Absolute (95% CI)		
1 [f]	randomised trials	serious [b]	not serious	not serious	very serious [c]	none	25	24	-	SMD **0.44 SD lower** (1.01 lower to 0.13 higher)	⨁◯◯◯ VERY LOW	CRITICAL

CI: Confidence interval; **SMD:** Standardised mean difference

Explanations

a. 1) Cristancho-Lacroix, 2015; 2) Núñez-Naveira, 2016
b. Serious concerns regarding risk of bias.
c. The sample size is <300 and effect estimate is imprecise.
d. 1) Cristancho-Lacroix, 2015; 2) Hattink, 2015.
e. Hattink, 2015
f. Cristancho-Lacroix, 2015

Table 4: Grade table for any internet-based information or education intervention + professional psychosocial support

Patient or population: Caregivers
Intervention: Technology (Internet: Information/Education + Professional psychosocial support)
Comparison: Control

Outcomes	Anticipated absolute effects* (95% CI)		Relative effect (95% CI)	№ of participants (studies)	Quality of the evidence (GRADE)	Comments
	**Risk with Control**	**Risk with Technology (Internet: Information/Education + Professional psychosocial support)**				
Change in Depression	-	SMD **0.34 SD lower** (0.63 lower to 0.05 lower)	-	180 (1 RCT) [a]	⊕⊕⊕◯ MODERATE [b]	Assessed using Center for Epidemiologic Studies Depression Scale: CES-D consisting of 20 items. The total score range is 0 to 60.
Change in Anxiety	-	SMD **0.36 SD lower** (0.66 lower to 0.07 lower)	-	180 (1 RCT) [a]	⊕⊕⊕◯ MODERATE [b]	Assessed using Hospital Anxiety and Depression Scale: HADS-A, 7-item anxiety subscale. The total score ranges from 0 to 21.

***The risk in the intervention group** (and its 95% confidence interval) is based on the assumed risk in the comparison group and the **relative effect** of the intervention (and its 95% CI).

CI: Confidence interval; **SMD:** Standardised mean difference

GRADE Working Group grades of evidence
High quality: We are very confident that the true effect lies close to that of the estimate of the effect
Moderate quality: We are moderately confident in the effect estimate: The true effect is likely to be close to the estimate of the effect, but there is a possibility that it is substantially different
Low quality: Our confidence in the effect estimate is limited: The true effect may be substantially different from the estimate of the effect
Very low quality: We have very little confidence in the effect estimate: The true effect is likely to be substantially different from the estimate of effect

Quality assessment							№ of patients		Effect		Quality	Importance
№ of studies	Study design	Risk of bias	Inconsistency	Indirectness	Imprecision	Other considerations	Technology (Internet: Information/Education + Professional psychosocial support)	Control	Relative (95% CI)	Absolute (95% CI)		
Change in Depression												
1 [a]	randomised trials	not serious	not serious	not serious	serious [b]	none	90	90	-	SMD **0.34 SD lower** (0.63 lower to 0.05 lower)	⨁⨁⨁◯ MODERATE	CRITICAL
Change in Anxiety												
1 [a]	randomised trials	not serious	not serious	not serious	serious [b]	none	90	90	-	SMD **0.36 SD lower** (0.66 lower to 0.07 lower)	⨁⨁⨁◯ MODERATE	CRITICAL

CI: Confidence interval; **SMD:** Standardised mean difference

Explanations
a. Blom, 2015
b. The sample size is <300.

Table 5: Grade table for any internet-based information or education + peer + professional psychosocial support

Patient or population: Caregiver
Intervention: Technology (Internet: Information/Education + Peer & Professional psychosocial support)
Comparison: Control

Outcomes	**Anticipated absolute effects*** (95% CI)		Relative effect (95% CI)	№ of participants (studies)	Quality of the evidence (GRADE)	Comments
	**Risk with Control**	**Risk with Technology (Internet: Information/Education + Peer & Professional psychosocial support)**				
Change in Depression	-	SMD **0.11 SD lower** (1.01 lower to 0.78 higher)	-	137 (3 RCTs) [a]	⨁◯◯◯ VERY LOW [b,c,d]	Assessed using Center for Epidemiologic Studies Depression Scale: CES-D consisting of 20 items. The total score range is 0 to 60.
Change in Stress / Distress	-	SMD **0.3 SD lower** (1.05 lower to 0.44 higher)	-	75 (2 RCTs) [e]	⨁◯◯◯ VERY LOW [b,d]	Assessed using Neuropsychiatric Inventory (NPI) with 12 domains on a 0 to 5 scale in one study and the degree of stress experienced on a 3-point severity scale in the other study.
Change in Overall Mental health	-	SMD **0.29 SD lower** (0.69 lower to 0.11 higher)	-	97 (1 RCT) [f]	⨁◯◯◯ VERY LOW [b,d]	Assessed using 16-item subset of negative mood items from the Short Version Profile of Mood States (SV-POMS). Likert-type items are rated on scales from 0–4.
Change in Quality of life	-	SMD **0.55 SD higher** (0.1 lower to 1.2 higher)	-	38 (1 RCT) [g]	⨁◯◯◯ VERY LOW [b,d]	Assessed using Quality of Life Scale, 16 item questionnaire; measuring six domains of QoL with a range of 16 to 112, higher scores indicate better QoL.

Table 5: Grade table for any internet-based information or education + peer + professional psychosocial support

Patient or population: Caregiver
Intervention: Technology (Internet: Information/Education + Peer & Professional psychosocial support)
Comparison: Control

Outcomes	Anticipated absolute effects* (95% CI)		Relative effect (95% CI)	№ of participants (studies)	Quality of the evidence (GRADE)	Comments
	**Risk with Control**	**Risk with Technology (Internet: Information/Education + Peer & Professional psychosocial support)**				
Change in Overall health	-	SMD **1.25 SD higher** (0.24 higher to 2.25 higher)	-	19 (1 RCT) [g]	⨁◯◯◯ VERY LOW [h]	Assessed using EuroQoL with 5 dimensions of QoL: mobility, self-care, usual activities, pain/discomfort, and anxiety/depression. The sub-scores can be combined to give a summary index value of 0-1.
Change in Coping	-	SMD **0.03 SD lower** (0.41 lower to 0.36 higher)	-	104 (1 RCT) [i]	⨁◯◯◯ VERY LOW [b,d]	Assessed using Brief Cope which was measured using two 5-point Likert-type scale items ranging from 0 (not at all) to 4 (a lot).

***The risk in the intervention group** (and its 95% confidence interval) is based on the assumed risk in the comparison group and the **relative effect** of the intervention (and its 95% CI).

CI: Confidence interval; **SMD:** Standardised mean difference

Table 5: Grade table for any internet-based information or education + peer + professional psychosocial support

Patient or population: Caregiver
Intervention: Technology (Internet: Information/Education + Peer & Professional psychosocial support)
Comparison: Control

Outcomes	**Anticipated absolute effects*** (95% CI)		Relative effect (95% CI)	№ of participants (studies)	Quality of the evidence (GRADE)	Comments
	**Risk with Control**	**Risk with Technology (Internet: Information/Education + Peer & Professional psychosocial support)**				

GRADE Working Group grades of evidence
High quality: We are very confident that the true effect lies close to that of the estimate of the effect
Moderate quality: We are moderately confident in the effect estimate: The true effect is likely to be close to the estimate of the effect, but there is a possibility that it is substantially different
Low quality: Our confidence in the effect estimate is limited: The true effect may be substantially different from the estimate of the effect
Very low quality: We have very little confidence in the effect estimate: The true effect is likely to be substantially different from the estimate of effect

Quality assessment							№ of patients		Effect		Quality	Importance
№ of studies	Study design	Risk of bias	Inconsistency	Indirectness	Imprecision	Other considerations	Technology (Internet: Information/Education + Peer & Professional psychosocial support)	Control	Relative (95% CI)	Absolute (95% CI)		
Change in Depression												
3 [a]	randomised trials	serious [b]	serious [c]	not serious	very serious [d]	none	66	71	-	SMD **0.11 SD lower** (1.01 lower to 0.78 higher)	⨁◯◯◯ VERY LOW	CRITICAL
Change in Stress / Distress												
2 [e]	randomised trials	serious [b]	not serious	not serious	very serious [d]	none	40	35	-	SMD **0.3 SD lower** (1.05 lower to 0.44 higher)	⨁◯◯◯ VERY LOW	CRITICAL
Change in Overall Mental health												

Quality assessment							№ of patients		Effect		Quality	Importance
№ of studies	Study design	Risk of bias	Inconsistency	Indirectness	Imprecision	Other considerations	Technology (Internet: Information/Education + Peer & Professional psychosocial support)	Control	Relative (95% CI)	Absolute (95% CI)		
1 [f]	randomised trials	serious [b]	not serious	not serious	very serious [d]	none	45	52	-	SMD **0.29 SD lower** (0.69 lower to 0.11 higher)	⨁◯◯◯ VERY LOW	CRITICAL
Change in Quality of life												
1 [g]	randomised trials	serious [b]	not serious	not serious	very serious [d]	none	18	20	-	SMD **0.55 SD higher** (0.1 lower to 1.2 higher)	⨁◯◯◯ VERY LOW	CRITICAL
Change in Overall health												
1 [g]	randomised trials	serious [h]	not serious	not serious	very serious [h]	none	9	10	-	SMD **1.25 SD higher** (0.24 higher to 2.25 higher)	⨁◯◯◯ VERY LOW	CRITICAL
Change in Coping												

Quality assessment							№ of patients		Effect		Quality	Importance
**№ of studies**	**Study design**	**Risk of bias**	**Inconsistency**	**Indirectness**	**Imprecision**	**Other considerations**	**Technology (Internet: Information/Education + Peer & Professional psychosocial support)**	**Control**	**Relative (95% CI)**	**Absolute (95% CI)**		
1 [i]	randomised trials	serious [b]	not serious	not serious	very serious [d]	none	49	55	-	SMD **0.03 SD lower** (0.41 lower to 0.36 higher)	⨁◯◯◯ VERY LOW	CRITICAL

CI: Confidence interval; **SMD:** Standardised mean difference

Explanations

a. 1) Pierce, 2009; 2) Smith, 2012; 3) Pagan-Ortiz, 2014.
b. Serious concerns regarding risk of bias.
c. The confidence intervals do not overlap across studies and statistical heterogeneity is high (I-squared = 83%; p = 0.002).
d. The sample size is <300 and effect estimate is imprecise.
e. 1) Marziali, 2006; 2) Torkamani, 2014.
f. DuBenske, 2014
g. Torkamani, 2014
h. Serious concerns for risk of bias and sample size <300.
i. Namkoong, 2012

Table 6: Grade table for any internet-based information or education + telephone and monitoring + peer + professional psychosocial support

Patient or population: Caregivers
Intervention: Technology (Internet + telephone: Monitoring + Peer & Professional psychosocial support)
Comparison: Control

Outcomes	**Anticipated absolute effects*** (95% CI)		Relative effect (95% CI)	№ of participants (studies)	Quality of the evidence (GRADE)	Comments
	**Risk with Control**	**Risk with Technology (Internet + telephone: Monitoring + Peer & Professional psychosocial support)**				
Change in Quality of life	-	SMD **0.6 SD lower** (1.31 lower to 0.11 higher)	-	32 (1 RCT) [a]	⨁◯◯◯ VERY LOW [b,c]	Quality of Life in Alzheimer's Disease, Informal caregivers filled-in 2 additional items about their overall judgment of their own quality of life. 15-items rated on a 4-point scale (range 15 to 60).

***The risk in the intervention group** (and its 95% confidence interval) is based on the assumed risk in the comparison group and the **relative effect** of the intervention (and its 95% CI).

CI: Confidence interval; **SMD:** Standardised mean difference

GRADE Working Group grades of evidence
High quality: We are very confident that the true effect lies close to that of the estimate of the effect
Moderate quality: We are moderately confident in the effect estimate: The true effect is likely to be close to the estimate of the effect, but there is a possibility that it is substantially different
Low quality: Our confidence in the effect estimate is limited: The true effect may be substantially different from the estimate of the effect
Very low quality: We have very little confidence in the effect estimate: The true effect is likely to be substantially different from the estimate of effect

Quality assessment							№ of patients		Effect		Quality	Importance
**№ of studies**	**Study design**	**Risk of bias**	**Inconsistency**	**Indirectness**	**Imprecision**	**Other considerations**	**Technology (Internet + telephone: Monitoring + Peer & Professional psychosocial support)**	**Control**	**Relative (95% CI)**	**Absolute (95% CI)**		
Change in Quality of life												
1 [a]	randomised trials	serious [b]	not serious	not serious	very serious [c]	none	17	15	-	SMD **0.6 SD lower** (1.31 lower to 0.11 higher)	⨁◯◯◯ VERY LOW	CRITICAL

CI: Confidence interval; **SMD:** Standardised mean difference

Explanations
a. Hattink, 2016
b. Serious concerns regarding risk of bias.
c. The sample size is <300 and effect estimate is imprecise.
